# Transcription factors and candidate functional SNPs associated with variation in fatty acid composition from skeletal muscle of pigs

**DOI:** 10.1111/age.70051

**Published:** 2025-10-11

**Authors:** Mariah C. Durval, Luiz F. Brito, Simara L. Fanalli, Artur O. Rocha, Lorena F. Benfica, Fernanda N. Ciconello, Camila S. Oliveira, Ingrid S. Garcia, Felipe A. Oliveira Freitas, Lucas E. Nascimento, Bruna P. Martins da Silva, Bárbara Silva‐Vignato, Aline S. M. Cesar

**Affiliations:** ^1^ Faculty of Animal Science and Food Engineering University of São Paulo Pirassununga São Paulo Brazil; ^2^ Department of Animal Sciences Purdue University West Lafayette Indiana USA; ^3^ Luiz de Queiroz College of Agriculture University of São Paulo Piracicaba São Paulo Brazil

**Keywords:** gene expression, lipid metabolism, Longissimus lumborum

## Abstract

Pork is an essential source of fatty acids (FAs) in the human diet. Fatty acids are important for various biological processes and can impact transcription regulation. The primary objective of this study was to identify candidate functional single nucleotide polymorphisms (SNPs) and expression quantitative trait loci (eQTL) associated with FA composition variation, and transcription factors (TFs) related to lipid metabolism using SNP array genotyping and Longissimus lumborum muscle transcriptome of Large White pigs. A total of 105 378 unique SNPs were identified, including 74 955 originating from RNA‐Seq data and 30 423 SNPs from the Porcine 50K SNP chip. These SNPs were tested for association with the skeletal muscle gene expression data (15 090 genes) using the matrixeqtl package. Genome‐wide association studies were conducted to test the association of these SNPs with FA trait variation, resulting in 74 254 eQTL, including 15 558 *cis*‐ and 58 696 *trans‐*eQTL. Furthermore, 23 eQTL hotspots were identified, along with four TFs related to lipid metabolism: *EGR1*, *SP1*, *CREB3* and *INSM*. The analysis identified two SNPs significantly associated with oleic and linolenic acids in the skeletal muscle of pigs. Candidate genes previously reported to influence meat quality in pigs and human health were identified, including *PITX3*, *NT5C2*, *FTL*, *GLIS1*, *API5* and *HILPDA*. Although these findings offer valuable insights into metabolic disease response and lipid metabolism, contributing to a better understanding of gene expression related to lipid metabolism, meat quality and FA composition in pigs, the limited sample size indicates that further validations using larger datasets are recommended.

## INTRODUCTION

Pork ranks among the most commonly produced and consumed meats in the world. Additionally, meat serves as a vital protein source for humans and directly influences human health (Food and Agriculture Organization, 2023; Guasch‐Ferré et al., 2015; Penkert et al., 2021; Wang et al., 2023). Furthermore, pigs are used as biomedical models for studying human diseases and disorders (Malgwi et al., 2022; Pan et al., 2021; Radcliffe et al., 2019). In recent years, there has been significant interest in improving the composition of fatty acids (FAs) in the muscle and adipose tissue of pigs to improve the pork's nutritional value (Criado‐Mesas et al., 2019; Minelli et al., 2023). The FA composition of intramuscular fat (IMF) significantly impacts pork's nutritional and sensory qualities (Almeida et al., 2021; Fanalli et al., 2022; Wood et al., 2008). For instance, monounsaturated FAs (MUFAs) are associated with greater oxidative stability of muscle than polyunsaturated FAs (PUFAs), enhancing the taste and color of meat (Wood et al., 2008). Furthermore, the consumption of unsaturated FAs has been associated with a reduced risk of cardiovascular diseases (Guasch‐Ferré et al., 2015; Safaei et al., 2024) compared with saturated FAs (SFAs). As a result, consumers are favoring pork with high IMF, which provides a more balanced FA composition in their diets and promotes more health‐conscious choices (Minelli et al., 2023; Puig‐Oliveras et al., 2016).

Biological mechanisms associated with the genetic basis of complex traits, such as meat quality and FA composition, can be elucidated based on techniques such as high‐throughput sequencing and gene expression analyses for the identification of expression quantitative trait loci (eQTL). Expression quantitative trait loci are DNA variants that regulate gene expression and contribute to the phenotypic variability of complex traits (Albert & Kruglyak, 2015; Cesar et al., 2018). Expression quantitative trait loci analyses enable the identification of genes whose expression levels are correlated with variants associated with traits of interest, while also providing detailed information on the biological mechanisms associated with their phenotypic variability (Albert & Kruglyak, 2015; Gamazon et al., 2019; Gerring et al., 2020).

Alongside eQTL analyses, genome‐wide association studies (GWASs) also contribute to elucidating biological processes underlying the variability in quantitative traits (Sahana et al., 2023). Furthermore, the integration of GWASs and eQTL analyses can contribute to identifying candidate causative genes, mutations or biological pathways related to traits of interest, as shown in previous studies focusing on meat quality in pig muscle (Longissimus dorsi) (Liu et al., 2021; Puig‐Oliveras et al., 2016). In recent years, GWASs have been conducted to discover the genetic foundations of FA composition traits in pigs (Revilla et al., 2018; Zhang et al., 2016). These studies have identified significant QTL associated with FAs and candidate genes that have functions directly relevant to FA metabolism.

The FA composition of pork can be modified through dietary changes (Fanalli et al., 2023; Mitchaothai et al., 2007), as well as through genetic selection (e.g. Zappaterra et al., 2020; Zhang et al., 2019). Various associations of genetic markers with phenotypic variability of complex traits have been identified based on GWASs with high‐density single nucleotide polymorphism (SNP) arrays (Sato et al., 2016; Sharmaa et al., 2015). The SNPs in these arrays are widely distributed throughout the genome, including genomic regions of introns, exons, promoters, enhancers and intergenic regions. The primary mode of action of these SNPs, which can affect gene expression, involves modifying transcription factor (TF) binding by either creating or disrupting TF binding sites (TFBSs) or altering the interaction strength of these regulatory factors with their specific sites (Degtyareva et al., 2021). Therefore, by binding at specific sites on DNA known as TFBSs, a TF directly interprets the regulatory segment of the genome, initiating the transcription process (Lambert et al., 2018; Merkulova et al., 2013). Hence, genetic variations within TFBSs can alter gene expression and, consequently, the phenotypic diversity of complex traits (Degtyareva et al., 2021; Inukai et al., 2017; Oksuz et al., 2023).

In a previous study using data from the same pig population, Almeida et al. (2021) observed that feeding pigs with diets containing different oil sources, such as soybean oil, canola oil and fish oil did not result in any significant changes in overall growth performance. However, significant differences were observed in the Warner–Bratzler shear force and hot dressing percentage. Furthermore, the same study showed that the FA composition of the intramuscular Longissimus lumborum (LL) muscle was modified owing to all types of dietary oils, leading to an increased PUFA content (Almeida et al., 2021). A previous eQTL study with the same population did not identify any significant eQTL related to carcass and body composition traits, but they were significantly enriched for many traits in the “Meat and Carcass” type QTL (Freitas et al., 2024). However, information on how genomic variants can influence the expression of genes in muscle associated with variation in FA composition using SNP chip genotyping and RNA‐Seq data in pigs is still limited. Therefore, the primary objective of this study was to identify candidate functional SNP, eQTL associated with FA composition variation in pigs and TFs related to lipid metabolism based on data from SNP genotyping and muscle transcriptome of Large White pigs.

## MATERIALS AND METHODS

### Ethics statement

This study was approved by the Animal Care and Use Committee from the Luiz de Queiroz College of Agriculture (ESALQ) from the University of São Paulo (Piracicaba, SP, Brazil; protocol number 2018.5.1787.11.6) and the Ethics Committee on Animal Use (number 2018‐28). All experimental procedures involving animals were performed based on the ethical principles outlined in the Guide for the Care and Use of Agricultural Animals in Agricultural Research and Teaching (Federation of Animal Science Societies, 2010).

### Animals

The experimental population was previously described by Fanalli et al. (2022, 2023). In brief, we selected 72 purebred immunocastrated Large White male pigs, the offspring of three sires and 32 Large White females. The animals were genotyped for the halothane mutation (*RYR1* gene), according to Fujii et al. (1991), and only the halothane homozygous negative (NN) pigs were included in the study. The average (standard deviation, SD) age of the pigs was 71 (1.8) days, with an average (SD) weight of 28.44 (2.95) kg. Pigs had *ad libitum* access to feed and water, provided through three‐hole dry self‐feeders and nipple drinkers in each pen, respectively. The experimental diets consisted of corn–soybean meal growing–finishing diets, supplemented with oil (Almeida et al., 2021). The pigs were slaughtered using electrical stunning in accordance with industry standards. The average (SD) weight of the pigs was 133.9 kg (9.4 kg), and they were 155 days old. The complete procedures are described in Almeida et al. (2021). Tissue samples were collected from the LL muscle immediately after slaughter, flash‐frozen in liquid nitrogen and stored at −80°C until total RNA extraction.

### Phenotypic data

We analyzed the following FAs: palmitoleic, oleic, linoleic, linolenic, MUFAs, PUFAs, omega‐3 (n‐3), and omega‐6 (n‐6). The FA composition determination was performed from the total lipid isolated from 100 g of the LL samples using the cold‐extraction method proposed by Bligh and Dyer (1959) and methylated according to the procedure outlined by the American Oil Chemists Society (method AM 5‐04) (Am, 2005). Additional details of the analyses are presented in Almeida et al. (2021). The descriptive statistics for each FA are presented in Table S1.

### RNA extraction, quality control, and statistical analyses

Total RNA was extracted from the LL muscle of 72 individuals, using the RNeasy® Mini Kit (Qiagen, Hilden, Germany), following the manufacturer's instructions. Total RNA quantification, purity and integrity were assessed using both Nanodrop 1000 and Bioanalyzer platforms. Each sample exhibited an RNA Integrity Number of 7 or higher. Library preparation was conducted using 2 μg of total RNA from each sample, following the protocol outlined in the TruSeq RNA Sample Preparation kit v2 guide (Illumina Inc., San Diego, CA, USA). The average size of libraries was determined using the Agilent Bioanalyzer 2100 (Agilent, Santa Clara, CA, USA), while quantification was performed using quantitative PCR with the KAPA Library Quantification kit (KAPA Biosystems, Foster City, CA, USA). The pooled samples were then sequenced across five lanes of a sequencing flow cell using the TruSeq PE Cluster kit v4‐cBot‐HS (Illumina, San Diego, CA, USA). Clustering and sequencing were carried out on the HiSeq2500 platform (Illumina, San Diego, CA, USA) with a TruSeq SBS Kit v4‐HS (200 cycles), following the manufacturer's instructions. All the sequencing analyses were performed at the ESALQ Genomics Center located in the Animal Biotechnology Laboratory at ESALQ–USP (Piracicaba, SP, Brazil).

The RNA‐Seq data underwent quality control (QC) steps using the trim galore package (version 0.6.5) (www.bioinformatics.babraham.ac.uk/projects/trim_galore). Reads with a minimum length of 70 bases and a Phred score higher than 33 were aligned and mapped to the pig reference genome (*Sus scrofa* 11.1) using the assembly available at Ensembl Release 102 (www.ensembl.org/Sus_scrofa/Info/Index). Data quality was checked using the fastqc software (version 0.11.8) (www.bioinformatics.bbsrc.ac.uk/projects/fastqc/). Alignment and mapping were performed using the star package (version 2.7.6a.) (Dobin & Gingeras, 2015).

### Identification of SNPs from RNA‐seq data

Variant calling analysis was conducted using the genome analysis toolkit (gatk, version 4.1.9.0) in the genomic variant call format (gvcf) mode (der Auwera et al., 2013; Franke & Crowgey, 2020). Genome coverage for each of the BAM files was calculated using the samtools (version 1.9) package (Danecek et al., 2021; Li et al., 2009). The addorreplacereadgroups tool from Picard (https://broadinstitute.github.io/picard/) was used to assign read group information. SplitNCigarReads was used to split reads that contain ‘*N*’ operations in their CIGAR string. Additionally, a recalibration of the quality scores was performed using gatk’s baserecalibrator followed by applybqsr. For variant calling, the haplotypecaller algorithm was applied to call variants individually, resulting in GVCF files for each sample. These files were then merged using the combinegvcf tool, and the joint genotyping analysis was performed using genotypegvcf, generating a VCF file with all the genotyped samples (der Auwera et al., 2013; Franke & Crowgey, 2020). Variant filtering was performed in multiple steps: SNPs were selected using the selectvariants function, and filters were applied using bcftools and vcftools to retain only variants with quality score ≥ 30, depth of coverage ≥ 30, minor allele frequency ≥ 0.05 and <20% missing data per site (max‐missing = 0.8).

The dataset used is available in the European Nucleotide Archive repository (EMBL‐EBI), under the accession no. PRJEB52629 (www.ebi.ac.uk/ena/data/view/PRJEB52629).

### Genotyping array data and quality control

DNA isolation and genotyping of the 72 samples were performed by geneseek (Neogen; Pindamonhangaba, SP, Brazil). The genotyping was based on the GeneSeek Genomic Profiler Porcine 50K chip containing 50915 SNPs. The SNPs were mapped to the *S. scrofa* genome (version 11.1) using the assembly available at Ensembl (Release 102). The SNP data from RNA‐Seq and 50K SNP chip were merged using the plink 1.9 software (Purcell et al., 2007). The merged dataset contained 112735 SNPs before QC (Table S2). bcftools (version 1.9; Danecek et al., 2021) was used to select variants based on SNP quality score ≥ 30 and total coverage depth ≥ 10 (Danecek et al., 2021; Li et al., 2009). The plink software (version 1.9) was used for QC. SNPs located on non‐autosomal chromosomes, with minor allele frequency <0.05, or call rate <0.95 were excluded from subsequent analyses. After QC, 105378 SNPs and 72 animals remained for further analyses, including 74 955 SNPs derived from RNA‐Seq and 30 423 SNPs from the 50K SNP chip.

### Identification of eQTL and hotspot regions

The eQTL analyses were conducted using the matrixeqtl package (Shabalin, 2012) from the r statistical program (version 4.3.0). matrixeqtl executes a separate test for each gene–SNP pair and corrects for multiple comparisons by calculating the false discovery rate (FDR) according to Benjamini and Hochberg (1995). The eQTL analyses enable the identification of SNPs that affect the expression levels of local (*cis‐*eQTL) or distant (*trans‐*eQTL) genes. For the analysis of *cis‐* and *trans‐*eQTL identification, genomic windows extending up to 1 Mb upstream from the start of the regulated gene and 1 Mb downstream from the end of the regulated gene were utilized to assess the cis (local) effects. Genomic windows more than 1 Mb away from the regulated gene were examined to assess distant effects (*trans*) (Freitas et al., 2024). Expression quantitative trait loci were tested between all possible pairs of genes (*n* = 15 090) and SNPs (*n* = 105 378). The analyses were done using the *modelLINEAR* (*linear*) function for both *cis‐* and *trans‐*eQTL based on the following fixed linear model:

G=β*s+PC+TR+ϵ

where **G** is the gene expression level in normalized transcripts per million (TPM), *β* is the SNP allelic substitution effect, *s* is the genetic marker covariate, coded as 0 (homozygous for the reference allele), 1 (heterozygous), or 2 (homozygous for the alternative allele) (Freitas et al., 2024), **PC** represents the first four principal components of the variance‐standardized relationship matrix (explaining 41.50% of the total variation) fitted as linear covariates to account for potential population stratification, **TR** is a dummy variable that represents the treatment effect, and *ϵ* is the random residual assumed to be i.i.d. *N* (0, *σ* ^2^) (Freitas et al., 2024). The principal component analysis was performed using the ‐pca function from the plink software (Purcell et al., 2007).

Linkage disequilibrium pruning analysis was initially performed considering an *r* ^2^ (linkage disequilibrium metric) threshold of 0.8, on the eQTL, using plink (version 1.90; Purcell et al., 2007). Subsequently, we identified eQTL hotspots, defined as markers that affect the gene expression level of many genes (Cesar et al., 2018). Herein, we considered eQTL hotspots as those affecting more than the average number of genes affected by all eQTL plus three times the SD, according to the criteria generally used to identify hub genes (Afonso et al., 2020).

### Functional annotation and functional enrichment analyses

The eQTL annotations were performed using the ensembl variant effect predictor (vep) tool (McLaren et al., 2016). The vep is a set of high‐performance tools for analyzing and annotating genomic variants in both coding and non‐coding genomic regions (McLaren et al., 2016). The reference genome assembly used was *S. scrofa* 11.1, available at Ensembl (www.ensembl.org/Sus_scrofa/Info/Index).

Functional enrichment analyses were performed using the database for annotation, visualization, and integrated discovery (david) (version 6.8) (Sherman et al., 2022). The list of genes regulating the *cis‐* and *trans‐*eQTL were applied separately. Ensembl gene IDs were uploaded to the gene functional classification tool from david, and then biological process (BP), cellular component (CC), molecular function (MF) and Kyoto Encyclopedia of Genes and Genomes (KEGG) pathways were selected for functional enrichment. Gene Ontology (GO) terms and KEGG pathways with a *p*‐value lower than 5% were considered significantly enriched.

The r package gallo (version 1.3; Fonseca et al., 2020) was used to analyze the QTL annotation and enrichment of the SNPs identified as *cis‐* and *trans‐*eQTL. For that, we used the AnimalQTL database (Hu et al., 2013) and considered a window of up to 100 kb downstream and upstream of the genomic coordinates of the *cis‐* and *trans‐*eQTL. The enrichment analysis was performed based on a hypergeometric test, implemented through the ‘qtl_enrich’ function in the gallo package (Fonseca et al., 2020). The plot_qtl_info function from GALLO was also utilized to illustrate the overview of QTL types and annotated traits. The graphs were generated using data for *cis‐* and *trans‐*eQTL separately.

### Motif and transcription factors discovery and refinement

Motif‐based sequence analyses were conducted using the MEME bioinformatics suite (version 5.5.3) (Bailey et al., 2009). A DNA motif is a short similar repeated pattern of nucleotides that holds biological significance (Jiang et al., 2013). This approach was conducted to identify common candidate motifs serving as TFBSs for TFs that control the expression of genes associated with IMF traits. Two meme suite analysis tools were used: streme (Bailey, 2021) and tomtom (Gupta et al., 2007).

streme, a motif discovery tool, was used to search for common motifs within a set of regulatory sequences. This tool identifies motifs enriched in the input sequences. To perform this analysis, streme compares the input sequences to a control dataset that is obtained by shuffling each of the input sequences (Bailey, 2021). The significant streme motifs identified were then assessed with the tomtom motif comparison tool to compare these motifs with known TFBSs. In the tomtom approach, sequences were compared to curated eukaryotic DNA provided by JASPAR vertebrates (Sandelin et al., 2004) and UniPROBE (Newburger & Bulyk, 2009) mouse databases of known TFBSs. The names of the TFs were selected according to the *S. scrofa* genome annotation, and the group of target genes consisted of genes regulated by the eQTL hotspots.

### Genetic modeling

A GWAS was performed to identify potential associations between SNP markers (and subsequently candidate genes harboring these SNPs) and the variability in FA traits (Table S3). We also evaluated the association between the gene expression level (at a FDR of 5%) and FA composition variation to identify additional candidate genes. Genetic parameters for palmitoleic, oleic, linoleic and linolenic acids, MUFAs, PUFAs, n‐3 and n‐6 FAs were estimated using the blupf90 suite of programs (Misztal et al., 2018) and Bayesian inference methods. The Gibbs sampling algorithm was executed with a chain of 500 000 cycles, burn‐in of 100 000 cycles, and thinning parameter of 100. The postgibbsf90 package (Misztal et al., 2018) was used to perform convergence diagnosis based on the Geweke method (Geweke, 1991) and calculate heritability (*h* ^2^), posterior means and posterior standard deviation (PSD). Another convergence diagnosis was also performed using the method described by Raftery et al. (1992), using the Bayesian Output Analysis (BOA) r package (version 1.1.8‐2; Smith, 2005). The SNP effects and *p*‐value estimates were obtained by back‐solving the gebv estimates using the blupf90 software (Misztal et al., 2018). The GWAS model can be described as:

y=Xb+Wu+e

where **y** is a vector of phenotypic records for each FA; **X** is an incidence matrix for the fixed effects (TR); **b** is a vector with solutions for fixed effects; **W** is the incidence matrix for random effects; and **u** is a vector with solutions for random effects, which were assumed as $u\approx N\left(0,{\mathrm{G\sigma}}_{u}^{2}\right)$, where G is a genomic relationship matrix, constructed based on the first method proposed by Vanraden (2008), and $\sigma_{u}^{2}$ is the additive genetic variance; **e** is a vector of residuals assumed $e\approx N\left(0,{\mathrm{I\sigma}}_{e}^{2}\right)$, where I is an identity matrix and $\sigma_{e}^{2}$ is the residual variance.

Significant eQTL with a unique genomic position (*n* = 14 399) were used for the association analyses with variation in palmitoleic, oleic, linoleic and linolenic acids, MUFAs, PUFAs, n‐3, and n‐6 FAs. The genomic inflation factor *λ* (or lambda value) for each GWAS analysis, which is expected to be 1 (Zhou & Stephens, 2014), was calculated using the ‘qchisq’ function in r (R Core Team, 2017).

## RESULTS

### Identification of eQTL

**FIGURE 1:**
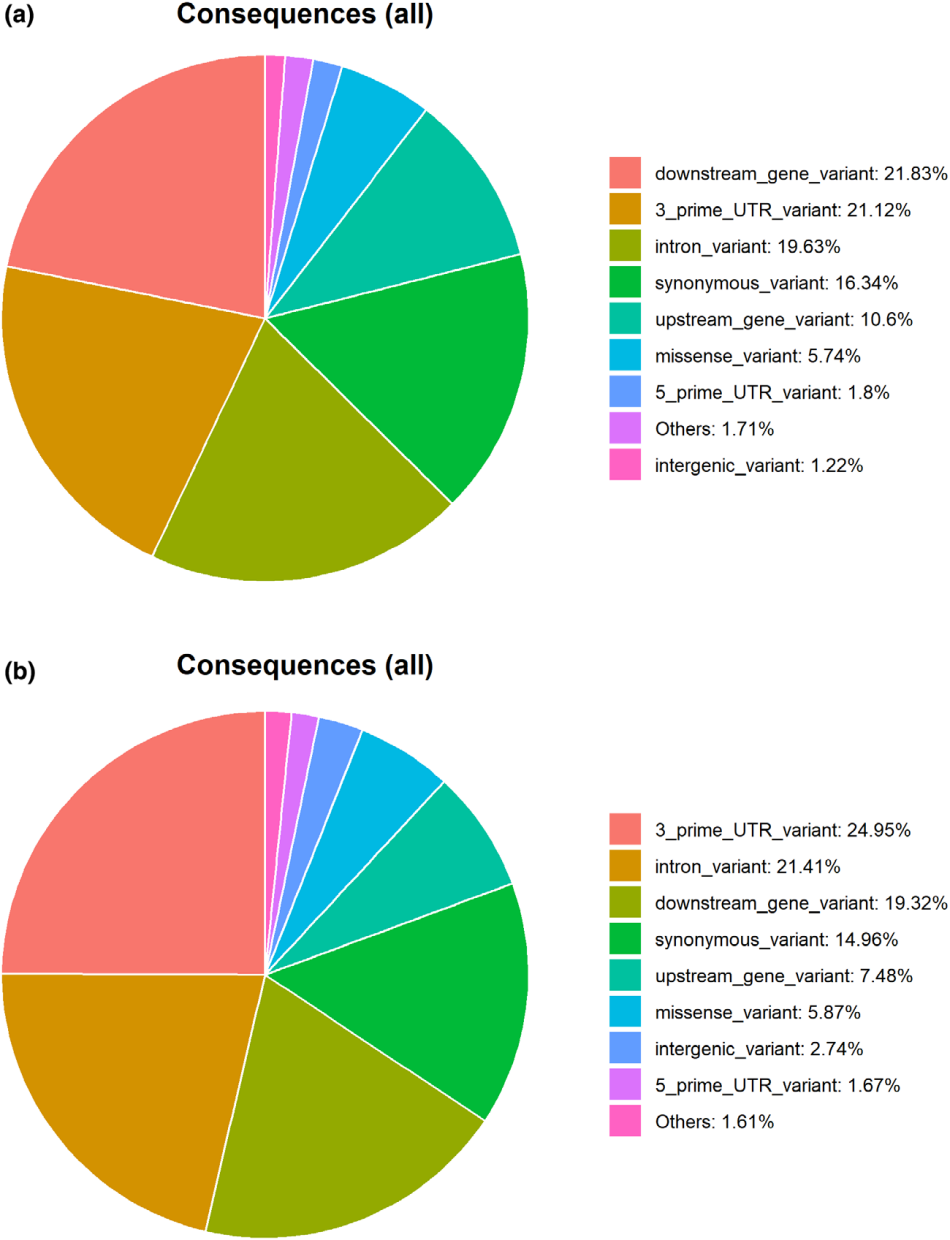
Variant annotation by the Ensembl variant effect predictor tool for *cis‐*expression quantitative trait loci (eQTL) (a) and *trans‐*eQTL (b).

A total of 74 254 eQTL were identified, of which 15 558 were classified as *cis‐*eQTL and 58 696 as *trans‐*eQTL. The *cis‐*eQTL regulated 1622 genes while the *trans‐*eQTL regulated 10 238 genes, with 1202 genes in common to both *cis‐*eQTL and *trans‐*eQTL. To ensure the reliability of the results and reduce the risk of false positives, the statistical analyses were corrected using the false discovery rate method, with a significance threshold set at 5% (FDR <0.05) (Benjamini & Hochberg, 1995). Based on the vep analyses, 40% of the *cis‐*eQTL were considered novel, and 60% as known variants. For the *trans‐*eQTL variants, 45.8% were considered novel and 54.2% as existing variants. The *cis‐*eQTL were located in downstream (21.83%), 3′ UTR (21.12%), intronic (19.63%) and upstream (10.6%) genomic regions (Figure 1a). The *trans‐*eQTL were located in 3′ UTR (24.95%), intronic (21.41%), downstream (19.32%) and upstream (7.48%) regions (Figure 1b). Analyses of the eQTL located in coding regions revealed that 75 and 25% of *cis‐*eQTL and 73 and 27% of *trans‐*eQTL were either synonymous (do not cause a change in protein sequence) or missense (cause a change in the protein sequence) variants (Cesar et al., 2018), respectively.

### Functional analyses of the eQTL

The GO enrichment analyses performed for *cis‐* and *trans‐*eQTL regulated genes showed 124 GO terms enriched for *cis‐*regulated genes and 465 GO terms for *trans‐*regulated genes (Tables 1 and 2).

**TABLE 1:** Top 10 significant gene ontologies (GO) for *cis‐*expression quantitative trait loci (eQTL) regulated genes using david software.

Category	Term	Number of genes	Adjusted *p*‐value
BP_DIRECT	GO:0072659: Protein localization to the plasma membrane	20	0.001
MF_DIRECT	GO:0046872: Metal ion binding	144	0.001
CC_DIRECT	GO:0005794: Golgi apparatus	66	0.002
BP_DIRECT	GO:0030336: Negative regulation of cell migration	14	0.002
CC_DIRECT	GO:0016020: Membrane	86	0.003
MF_DIRECT	GO:0046527: Glucosyltransferase activity	4	0.003
BP_DIRECT	GO:0045742: Positive regulation of epidermal growth factor receptor signaling pathway	5	0.004
CC_DIRECT	GO:0005813: Centrosome	44	0.004
BP_DIRECT	GO:0009306: Protein secretion	9	0.004
MF_DIRECT	GO:0030674: Protein binding, bridging	11	0.005

*Note*: The label ‘Category’ denotes the classification of gene ontology terms, which are categorized into three main categories: biological process (BP), cellular component (CC) and molecular function (MF). The label ‘Term’ describes the function or role of the genes grouped under each category. The label ‘Number of genes’ refers to the number of genes in the GO category. The label ‘adjusted *p*‐value’ represents the statistical significance of the enrichment of genes associated with the gene ontology term.

**TABLE 2:** Top 10 significant gene ontologies (GOs) for *trans‐*eQTL regulated genes by using david software.

Category	Term	Number of genes	Adjusted *p*‐value
BP_DIRECT	GO:0060271: Cilium assembly	93	0.001
BP_DIRECT	GO:0001525: Angiogenesis	82	0.001
BP_DIRECT	GO:0046777: Protein autophosphorylation	82	0.001
CC_DIRECT	GO:0005777: Peroxisome	49	0.001
CC_DIRECT	GO:0071007: U2‐type catalytic step 2 spliceosome	21	0.001
BP_DIRECT	GO:0007229: Integrin‐mediated signaling pathway	64	0.001
MF_DIRECT	GO:0004725: Protein tyrosine phosphatase activity	68	0.001
CC_DIRECT	GO:0000940: Condensed chromosome outer kinetochore	13	0.001
BP_DIRECT	GO:0043087: Regulation of GTPase activity	45	0.001
MF_DIRECT	GO:0042826: Histone deacetylase binding	55	0.001

*Note*: The label ‘Category’ denotes the classification of gene ontology terms, which are categorized into three main categories: biological process (BP), cellular component (CC) and molecular function (MF). The label ‘Term’ describes the function or role of the genes grouped under each category. The label ‘Number of genes’ refers to the number of genes in the GO category. The label ‘adjusted *p*‐value’ represents the statistical significance of the enrichment of genes associated with the gene ontology term.

The KEGG pathway enrichment analyses indicated that the *cis‐* and *trans‐*eQTL regulated genes were significantly enriched in 15 (Table S4) and 206 (Table S5) KEGG pathways, respectively. Table 3 shows these pathways associated with *trans‐*eQTL, in which the KEGG functional analysis revealed are enriched pathways related to FAs and lipids. However, no pathway associated with *cis‐*eQTL was directly related to FAs and lipids. This highlights the role of *trans‐*eQTL in important pathways related to FA metabolism, lipolysis and diseases.

**TABLE 3:** The main pathways associated with fatty acids (FAs) identified in the enrichment analysis of *trans‐*eQTL regulated genes.

Term	Count	Adjusted *p*‐value
ssc04071: Sphingolipid signaling pathway	90	<0.001
ssc05417: Lipid and atherosclerosis	135	<0.001
ssc01212: Fatty acid metabolism	43	<0.001
ssc04923: Regulation of lipolysis in adipocytes	40	<0.001
ssc04920: Adipocytokine signaling pathway	47	0.002
ssc01040: Biosynthesis of unsaturated fatty acids	22	0.005
ssc00062: Fatty acid elongation	20	0.006
ssc00071: Fatty acid degradation	29	0.013
ssc00561: Glycerolipid metabolism	38	0.023

*Note*: The label ‘Term’ lists the names of the KEGG pathways related to fatty acid and lipid metabolism. The label ‘Count’ indicates the number of genes involved in each KEGG pathway. The label ‘adjusted *p*‐value’ represents the statistical significance for the enrichment of each KEGG pathway.

**FIGURE 2:**
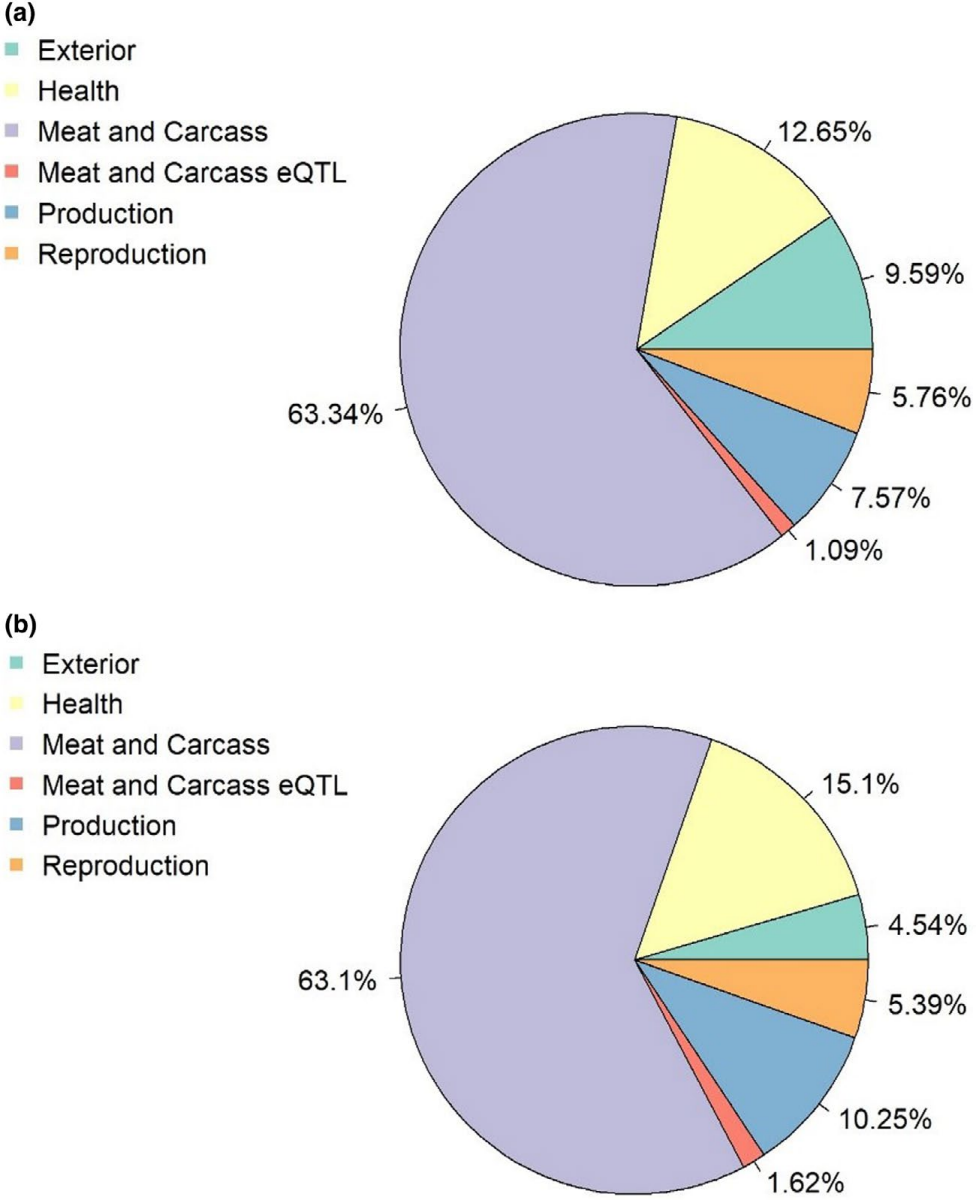
(a) Percentage of quantitative trait loci (QTL) type for *cis‐*eQTL. (b) Percentage of QTL type for *trans‐*eQTL.

The QTL annotation analysis for *cis‐* and *trans‐*eQTL resulted in 63.34 and 63.10% QTL in the ‘Meat and carcass’ QTL classes. Meanwhile the second highest percentage was for ‘Health’, with 12.65 and 15.10% for *cis‐* and *trans‐*eQTL, respectively (Figure 2a,b).

### Hotspot regulatory polymorphisms

**FIGURE 3:**
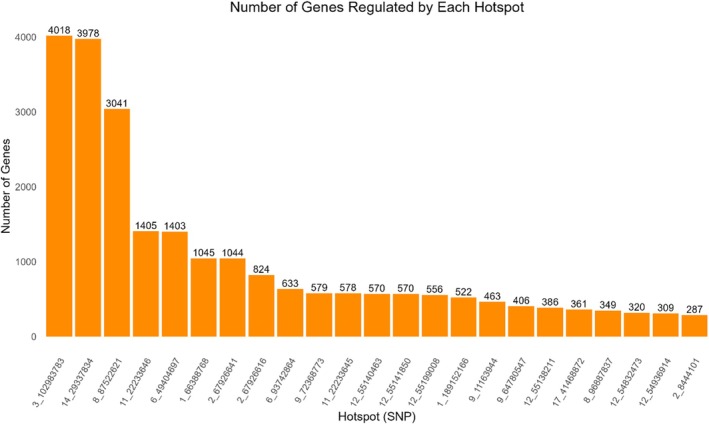
Histogram showing the number of genes regulated by each expression quantitative trait loci (eQTL) hotspot.

Variants that impact the expression of several genes (eQTL hotspots) have the potential to modulate metabolic pathways and cause phenotypic variability in traits of interest (Albert & Kruglyak, 2015). Based on this definition, we defined hotspots as those polymorphisms associated with the expression levels of 277 or more genes (those affecting more than the average of genes affected by all eQTL plus three times the SD, mean = 6.28; SD = 90.31; threshold = 277.21) (Table S6). We identified 23 hotspots distributed along the *S. scrofa* chromosomes (SSC), including two hotspots located on SSC1 (66 388 768 and 89 152 166 bp), three hotspots on SSC2 (67 926 641, 67 926 616, and 8 444 101 bp), one hotspot on SSC3 (102 983 783 bp), two hotspots on SSC6 (49 404 697 and 93 742 864 bp), two hotspots on SSC8 (87 522 621 and 96 887 837 bp), three hotspots on SSC9 (72 368 773, 11 163 944, and 4 780 547 bp), two hotspots on SSC11 (22 233 646 and 22 233 645 bp), six hotspots on SSC12 (55 140 463, 55 141 850, 55 199 008, 55 138 211, 54 832 473, and 54 936 914 bp), one hotspot on SSC14 (29 337 834 bp) and one hotspot on SSC17 (41 468 872 bp). The hotspot eQTL on SSC3:102983783 regulated the highest number of genes (*n* = 4018), and the hotspot eQTL on SSC2:8444101 regulated the lowest number of genes (*n* = 287) (Figure 3). In the vep analysis, it was identified that among these 23 hotspots, the SSC14 (29 337 834 bp) and SSC12 (55 140 463 bp) hotspots are missense variants. Genes such as Lipase E, Hormone Sensitive Type (*LIPE*), Fatty Acid Synthase (*FASN*), Acetyl‐CoA Carboxylase Alpha (*ACACA*), Acyl‐CoA Oxidase 1 (*ACOX1*), Diacylglycerol o‐Acyltransferase 1 (*DGAT1*), Lipase G, Endothelial Type (*LIPG*), Fatty Acid Binding Protein 4 (*FABP4*), Fatty Acid Binding Protein 3 (*FABP3*), Fatty Acid Binding Protein 5 (*FABP5*) Phospholipase A2 Group VII (*PLA2G7*), and Perilipin 2 (*PLIN2*) are related to lipid metabolism and FA production in pigs and many other species (Deng et al., 2023; Malgwi et al., 2022; Villaplana‐Velasco et al., 2021; Wang, Wang, et al., 2020; Yan et al., 2025), and were among the regulated genes.

### Common candidate motifs and lipid‐associated TFs in swine

The putative promoter regions of the hotspots were examined for the presence of TFBSs using the streme tool (Bailey, 2021). This analysis uncovered 615 significantly enriched motifs, based on an *E*‐value threshold of 0.05 (Figure S1). Subsequently, we used the tomtom (Gupta et al., 2007) tool to compare these identified motifs with known TFBSs documented in the literature. The KEGG pathway analysis, using the TFs identified by the tomtom tool, revealed four IMF‐correlated genes including *Early growth response factor 1* (*EGR1*), *Sp1 Transcription Factor* (*SP1*), *CAMP responsive element binding protein 3* (*CREB3*) and *Insulinoma‐associated protein 1* (*INSM1*).

### Genetic background of FA composition

An association analysis was performed with the 14 399 eQTL distributed along the 18 autosomal chromosomes in pigs. Moderate to high heritability estimates were obtained for all FAs, including palmitoleic acid (0.47 ± 0.10), oleic acid (0.62 ± 0.20), linoleic acid (0.61 ± 0.20), linolenic acid (0.24 ± 0.10), SFAs (0.41 ± 0.20), MUFAs (0.64 ± 0.20), PUFAs (0.64 ± 0.20), n‐3 (0.43 ± 0.20), and n‐6 (0.62 ± 0.20), as presented in Table 4. Although the sample size for the analyses was small (i.e. high PSD), all the variance components converged based on the convergence criteria used.

**TABLE 4:** Heritability (*h* ^2^) estimates for the analyzed traits with posterior standard deviation and highest posterior density.

Trait	*h* ^2^	PSD	HPD interval (95%)
Palmitoleic acid	0.47	0.19	0.09–0.83
Oleic acid	0.62	0.22	0.22–0.99
Linoleic acid	0.61	0.21	0.24–0.99
Linolenic acid	0.24	0.19	0.0001–0.62
Saturated fatty acids (SFAs)	0.41	0.25	0.0007–0.86
Monounsaturated fatty acids (MUFAs)	0.64	0.21	0.24–0.99
Polyunsaturated fatty acids (PUFAs)	0.64	0.21	0.27–0.99
Omega‐6	0.43	0.23	0.02–0.86
Omega‐3	0.62	0.21	0.24–0.99

Abbreviations: *h* ^2^, heritability; HPD, highest posterior density interval (95%) lower and upper bounds of mean; PSD, posterior standard deviation.

Two SNPs were significantly associated with FA composition variation (Table 5). The SNP SSC14:113843655 was associated with oleic acid variation and regulated the expression of four genes in *cis‐* and 20 genes in *trans‐*, distributed across chromosomes SSC1, SSC2, SSC4, SSC6, SSC7, SSC8, SSC13, SSC14, SSC17 and SSC18. The SNP SSC8:38763511 was significantly associated with linolenic acid variation and the expression of one gene (*SLAIN2*) regulated by a *cis‐*eQTL.

**TABLE 5:** Genome‐wide association study‐based SNPs associated with fatty acid composition.

SNP ID	Chromosome	Position	Fatty acid affected	*p*‐Value	Genes regulated (*cis*/*trans*)
SSC14:113843655	SSC14	113 843 655	Oleic acid	<0.001	*cis*: *PITX3*, *NFKB2*, *NT5C2*, *TAF5*; *trans*: *SCAMP2*, *FTL*, *GLIS1*, *PLRG1*, *LSMEM2*, *GRIP2*, *API5*, *UTP15*, *NUS1*, *SEC22B*, *RBM22*, *YAE1*, *HILPDA*, *TOR1A*, *ATP6V0B*, *CAMK2B*
SSC8:38763511	SSC8	38 763 511	Linolenic acid	Significant	*cis*: *SLAIN2*

*Note*: The label ‘SNP ID’ refers to the identifier and genomic position of the significant single nucleotide polymorphism. ‘Chromosome’ and ‘Position’ indicate the genomic coordinates of the SNP. ‘Fatty acid affected’ specifies the fatty acid whose variation was significantly associated with the SNP. ‘*p*‐Value’ represents the statistical significance of the association. ‘Genes regulated (*cis*/*trans*)’ lists the genes whose expression is regulated by the SNP, classified as *cis* or *trans*.

## DISCUSSION

### Identification of eQTL

The association analyses between each of the 105 378 SNPs and the expression level of 15 090 genes expressed in pig skeletal muscle considered only SNPs deemed significant based on a 5% FDR threshold (Benjamini & Hochberg, 1995). This approach led to the identification of 15 558 *cis‐*eQTL and 58 696 *trans‐*eQTL that were regulating 1622 and 10 238 genes, respectively. These results align with findings from previous eQTL studies, further supporting the robustness of our approach (Carmelo & Kadarmideen, 2020; Freitas et al., 2024; Võsa et al., 2021; Wang et al., 2024). The higher number of *trans‐*eQTL compared with *cis‐*eQTL was expected, as our SNP panel includes a significant proportion of variants located in intronic and intergenic regions, which are typically farther from genes. *Cis* regulators usually impact only a limited number of genes, while *trans* regulators can have pleiotropic effects on multiple genes (Zande et al., 2022). Moreover, understanding *trans‐*eQTL can help us to identify the distal effects of a single variant as well as gene regulation mechanisms (Võsa et al., 2021).

### Functional analyses of the eQTL

Annotation and functional enrichment analyses were conducted separately for *cis‐* and *trans‐*eQTL. This approach was taken because *cis‐* and *trans‐*eQTL are typically involved in distinct mechanisms for regulating gene expression (Pierce et al., 2014). *Cis*‐eQTL acts on genes close to where they are found. *Trans*‐eQTL, on the other hand, regulates the expression of distant genes. Annotation analysis revealed that *cis‐* and *trans‐*eQTL have missense variants located on coding regions, suggesting that both *cis‐* and *trans‐*eQTL are functionally relevant and can affect the sequence and function of proteins. In addition, the hotspots on SSC14 (29 337 834 bp) and SSC12 (55 140 463 bp) are missense variants. The hotspot on SSC14 (29 337 834 bp) regulates 3041 genes while the one on SSC12 (55 140 463 bp) regulates 570 genes. This suggests that these missense variant hotspots are influencing the expression of numerous genes and are in coding regions that result in changes to the amino acid sequence of the proteins. This information could provide valuable insights for future studies on how changes in gene expression are related to alterations in protein structure and function, which may have implications for understanding genetic diseases and other complex traits.

The highest percentage of *trans‐*eQTL is in the 3′‐untranslated region (3′ UTR). SNPs located in the 3′ UTR of genes can affect mRNA stability and translation by regulating miRNA–mRNA interaction (Liu et al., 2019). Consequently, SNPs within the miRNA binding site can reduce the miRNA binding capacity, thus being associated with diseases (Moszyńska et al., 2017; Pirooz et al., 2018; Roy & Mallick, 2017). SNPs in miRNA binding sites can also influence economically important traits in animals, including pigs, such as meat color (Wang, Shen, et al., 2022), fat deposition (Shao et al., 2011), litter size (Lei et al., 2011) and reproductive performance (Ma et al., 2016). The highest percentages of *cis‐*eQTL were located on downstream genes. SNPs harbored in this region can influence gene expression by modulating post‐transcriptional regulatory mechanisms, as well as mRNA stability, translation efficiency and nuclear export (Fan et al., 2025; Liao & Lee, 2010; Shulman & Elkon, 2020).

A high percentage of QTL categorized in the ‘Meat and Carcass’ group of traits is probably due to the overrepresentation of studies in pigs evaluating carcass and meat quality traits. Another important reason is that consumers associate the composition of FAs in muscles with meat quality. Specifically, higher levels of SFAs and MUFAs are related to greater meat acceptance, while increased levels of PUFAs are associated with lower consumer acceptance (Cameron et al., 2000; Świątkiewicz et al., 2021). The second QTL trait category found herein was ‘Health’. In pigs, FAs, particularly SFAs with 6–12 carbons, are beneficial owing to their antibacterial and antiviral properties (Jackman et al., 2022). Furthermore, these FAs can enhance feed digestibility, improve feed efficiency and contribute to the healthy growth of pigs (Baltić et al., 2017; Jackman et al., 2020; Messens et al., 2010; Thormar & Hilmarsson, 2007). From a nutritional perspective, excessive consumption of SFAs, particularly myristic and palmitic acids, may potentially elevate the likelihood of cardiovascular disease and type 2 diabetes in humans (Calder, 2015; Rasmussen et al., 2006; Zhang et al., 2019). Quantitative trait locus enrichment analyses were performed to account for this potential bias of reported results in the literature by fitting a hypergeometric test based on the significance of the eQTL.

We identified eQTL associated with the *ACACA*, *FASN* and *LIPE* genes, which are well‐known candidate genes for fatty acid composition in pigs. The *ACACA* and *FASN* genes play important roles in lipogenesis and the biosynthesis of fatty acids in pigs (Crespo‐Piazuelo et al., 2020; Óvilo et al., 2022; Poklukar et al., 2023). Moreover, *FASN*, *ACACA* and *LIPE* are implicated in fat deposition through the balance between lipogenesis and lipolysis (Óvilo et al., 2022). The *LIPE* gene, responsible for encoding a hormone‐sensitive lipase, contributes to triacylglycerol biosynthesis (Recazens et al., 2021). Zappaterra et al. (2016) revealed a positive association of LIPE expression with IMF in pigs.

We also identified additional relevant genes. *PLA2G7* is involved in the transport of oleic acid, an essential FA that contributes to pork quality (Villaplana‐Velasco et al., 2021). The *FABP3*, *FABP4* and *FABP5* genes are implicated in fat related traits in pigs, particularly in association with higher FA and IMF content (Ballester et al., 2017; Estellé et al., 2006; Villaplana‐Velasco et al., 2021). This gene family (*FABP3*, *FABP4*, and *FABP5*) has been highlighted for its role in the variation of fat deposition among pig lines (Puig‐Oliveras et al., 2014; Villaplana‐Velasco et al., 2021). Additionally, studies have suggested that *LIPG* and *PLIN2* may contribute to the regulation of subcutaneous fat deposition (Yan et al., 2025).

### Transcription factors

Transcription factors control gene expression by binding to particular DNA regions (Spitz & Furlong, 2012). Numerous TFs serve as ‘master regulators’, controlling processes that determine cell types and developmental patterning, and the regulation of specific metabolic pathways (Lambert et al., 2018; Lee & Young, 2013). The occurrence of polymorphisms in TFs and TF‐binding sites underlies numerous human diseases (Huo et al., 2019; Lambert et al., 2018). A single TF can influence several genes in distinct cell types (Lambert et al., 2018). The search for significant TF binding sites in the promoter regions of the genes in hotspots can represent a powerful strategy for identifying key regulators of complex biological processes. Finding promoter regions is important for the comprehension of gene expression regulation mechanisms.

Meat is the primary source of lipids in the human diet, with high nutritional value and rich in SFAs (Geiker et al., 2021; Wood et al., 2008). However, meat has a high level of saturated fat associated with metabolic diseases, such as obesity, diabetes and cardiovascular diseases (Bell & Culp, 2022; Julibert et al., 2019; Lin et al., 2004). Studying animal models of human diseases contributes to understanding the mechanisms implicated in disease pathogenesis. This knowledge provides the necessary resources for developing gene therapies to cure these diseases or conditions in humans. Pigs have served as a biomedical model to study diseases such as diabetes, which are becoming increasingly important as many countries struggle with major obesity‐related problems (Larsen & Rolin, 2004; Naqvi et al., 2023; Wolf et al., 2014). In this way, it is essential to understand how gene expression acts on FAs and metabolic pathways. Four TFs were annotated within the eQTL hotspots identified in our study: *EGR1*, *SP1*, *INSM1* and *CREB3*. All of these TFs have been previously implicated in lipid metabolism (Corominas et al., 2015; Khan et al., 2019; Wang et al., 2017). A study conducted on pig muscle demonstrated the relationship between *EGR1* and lipid biosynthesis (Wang et al., 2017). Another study on pigs showed that *SP1* is involved in adipogenesis (Corominas et al., 2015). Furthermore, research indicated that the transcription factor *INSM1* regulates important genes associated with adipocyte differentiation in cattle (Khan et al., 2019). *CREB3* belongs to a TF family linked to several metabolic pathways, including lipid and cholesterol metabolism, as well as glucose regulation (Gao et al., 2024; Khan & Margulies, 2019).

The *TF Early Growth Response protein 1* (*EGR1*) belongs to the family of zinc finger TFs and is related to cell growth, differentiation and function (Banerji & Saroj, 2021). *EGR1* is associated with adipocyte biogenesis, as well as with lipid metabolic processes and deposition (Faggion et al., 2023). In addition, *EGR1* has been linked to variations in both fat thickness and IMF content (Cesar et al., 2018; Faggion et al., 2023). The TF *EGR1* participates in the KEGG pathway ssc04933: AGE‐RAGE signaling pathway in diabetic complications. *EGR1* plays a regulatory role in insulin and cholesterol biosynthesis (Meriin et al., 2022; Shen et al., 2011). Another study on Longissimus dorsi in Laiwu pigs reported the significance of the TF *EGR1* in fat metabolism and lipid biosynthesis (Wang et al., 2017). In a human study, two variations in *EGR1* were associated with altered lipid metabolism (Brand et al., 2000; Meriin et al., 2022). *EGR1* has also been reported to have a negative effect on adipose differentiation (Boyle et al., 2009). Corroborating our study, it is suggested that *EGR1* TF regulates lipid metabolism.

The *TF Specificity Protein 1* (*SP1*) was also found in a diabetes‐related pathway (KEGG pathway ssc05415: Diabetic cardiomyopathy). *SP1* is a TF that controls the expression levels of various genes, including genes associated with angiogenesis. High *SP1* expression has been associated with angiogenesis (Yao et al., 2004). Alterations in the *SP1* level may contribute to vascular complications associated with diabetes (Beishline & Azizkhan‐Clifford, 2015; Cabrera et al., 2023). A study in pigs showed that TF *SP1* is implicated in the mechanism of lipogenic genes, like *ELOVL Fatty Acid Elongase 6* (*ELOVL6*) (Corominas et al., 2015). In another study involving Hezuo Tibetan pigs, TF *SP1* was shown to be an important transcription regulator of the *Heme Oxygenase 1* (*HMOX1*) gene (Wang, Yang, et al., 2020). Polymorphisms in the *HMOX1* gene have been associated with a higher risk of metabolic disorders (Martínez‐Hernández et al., 2015; Meng et al., 2021). Based on this information, TF *SP1* may be associated with the regulation of numerous genes involved in metabolic disorders, highlighting its important role in these conditions.

The TF *CREB3* participates in KEGG pathway ssc04927: Cortisol synthesis and secretion. *CREB3* is a TF involved in stress regulation, which affects the hypothalamic–pituitary–adrenal axis activity in mice (Larson et al., 2021). The TF *CREB3* was previously associated with the variability in stress responsiveness in pigs. Variations in cortisol levels occur in response to both physiological and psychological stressors (James et al., 2023). Elevated cortisol levels have been causally associated with fat deposition and weight increase, as glucocorticoids facilitate the transformation of preadipocytes into mature adipocytes (Incollingo Rodriguez et al., 2015). Polymorphisms in the *CREB3* gene can potentially influence the stress response in pigs, including altering gene expression through promoter/enhancer activity regions (Larson et al., 2021).

The TF *INSM1* is involved in the KEGG pathway ssc04350: TGF‐*β* signaling pathway. TGF‐*β* signaling exerts a range of effects on the development, proliferation, apoptosis, dedifferentiation, and function of islet *β* cells (Lee, Lee, & Rane, 2021). The synthesis and secretion of insulin to decrease blood glucose levels in adults are primarily carried out by islet *β* cells (Papa, 2012). Dysfunction or loss of these *β* cells significantly contributes to the development of diabetes (Cerf, 2013). In cases of metabolic syndrome and diabetes, there is a systemic activation of TGF‐*β* signaling as the serum levels of TGF‐*β*1 are higher in both obese patients and animal models of diabetes (Wang, Wang, et al., 2022). *INSM1* regulates the chromatin‐modifying factors involving histone deacetylases, which are fundamental to various biological processes, including gene expression and regulation (Zhang et al., 2012). *INSM1* plays a key role in regulating target genes or indirectly influencing growth factor signaling pathways (Zhang et al., 2014). The TF *INSM1* has been reported as a regulator of the *TOR complex 2* (*TORC2*) gene in cattle, which subsequently serves as a positive controller of bovine adipocyte proliferation and differentiation (Khan et al., 2019).

Hotspots occur when a single polymorphism induces generalized downstream alterations in the expression of distant genes, all mapped to the same genomic locus. Numerous genetic polymorphisms, especially in TFs, could result in substantial biological effects that could be observable as eQTL hotspots (Breitling et al., 2008). This study found four TFs (*EGR1*, *SP1*, *CREB3* and *INSM1*) in eQTL hotspots previously described for their association with lipid metabolism. Thus, the TFs found in this study, *EGR1*, *SP1*, *CREB3*, and *INSM1*, may regulate genes involved in lipid metabolism and are involved in important pathways related to metabolic diseases. Our findings improve our understanding of the genetic mechanisms underlying gene expression regulation in FA metabolism in muscle. In general, the expression of genes associated with FA and lipid metabolism is complexly regulated. GWAS was carried out to better understand the relationship between the eQTL identified in these studies and FAs.

### Genetic background of fatty acid composition

Expression quantitative trait locus and GWAS analyses offer a direct view of the characterization of genes that have genetic variants that modulate expression levels. In this study, 74 254 eQTL were used for the association analyses, including 58 696 *trans‐*eQTL and 15 558 *cis‐*eQTL. The heritability estimates for the traits evaluated ranged from 0.25 to 0.67, which are consistent with literature reports (Fernández et al., 2003; Sellier, 1998; van Son et al., 2017) and indicate that selection for improved FA composition is feasible (Gjerlaug‐Enger et al., 2011; van Son et al., 2017). Only linolenic acid, with a heritability value of 0.24, was below the heritability range values for FA composition (0.25–0.67) previously reported in studies on pigs (Fernández et al., 2003; Sellier, 1998; van Son et al., 2017). Moreover, palmitic acid (0.47), oleic acid (0.62), and linoleic acid (0.61) heritability estimates were higher than those reported by Fernández et al. (2003), with values of 0.31, 0.30, and 0.29. It is worth noting that a small sample size was used for the analyses, which can affect the accuracy of the estimates. Although the heritability values are generally in line with previous findings (Fernández et al., 2003; Sellier, 1998; van Son et al., 2017), it is important to note that the PSDs were higher, which indicate less precise estimates than other literature reports. Future studies using larger datasets are necessary to validate these findings.

Proper GWASs require a large number of individuals with both genomic and phenotypic records to achieve sufficient statistical potential (Hong & Park, 2012; Spencer et al., 2009). Numerous studies have emphasized that GWASs conducted with small sample sizes exhibit limited statistical ability to detect loci with small effects, i.e. only loci with very large effects are likely to achieve the genome‐wide significance threshold when small sample sizes are used (Davenport et al., 2015; Huo et al., 2019). Our sample size is substantially lower than in other studies (Lee, Kang, et al., 2021; Sanjari Banestani et al., 2023). This factor might have affected our ability to detect significant associations. In addition to the small sample size, another limiting factor was the utilization of a maternal line breed (Large White) instead of a terminal sire breed (e.g. Duroc) that has been more intensively selected for meat quality traits. As genetic polymorphisms are population specific and this study was conducted using Large White pigs, the findings may not be directly applicable to other breeds. Future research should investigate breed‐specific differences to determine the applicability of these findings to other pig breeds, especially terminal‐sire breeds.

The FA composition of pork can influence meat quality and consequently, human health. Notably, the prevalence of MUFAs in skeletal muscle, particularly oleic acid, which constitutes approximately 40% of the FAs present in pork tissue, has been reported to have a positive association with overall palatability factors such as flavor, juiciness and tenderness in pork (Hwang & Joo, 2017; Światkiewicz et al., 2016).

The SNP significantly associated with oleic acid variation was SSC14:113843655. This SNP is associated with genes expressed in muscle, *PITX3* and *NT5C2*, which play important roles in FA and lipid metabolism (Li et al., 2021; Palombo et al., 2018). These genes are in a *cis‐*eQTL region, indicating that nearby polymorphism may regulate their expression. *PITX3* is essential for the myogenic differentiation of muscle satellite cells (Knopp et al., 2013). A recent study on Duroc pigs demonstrated that the *PITX3* gene is important for lipid metabolism and muscle development (Li et al., 2021). *The 5′*‐*Nucleotidase*, *Cytosolic II* (*NT5C2*) gene is also expressed in skeletal muscle and is associated with FA composition (Palombo et al., 2018). *NT5C* is involved in inhibiting basal lipid oxidation and glucose transport in skeletal muscle (Kulkarni et al., 2011).

The QTL 14:113843655 was also associated with genes located in a *trans‐*eQTL region, *FTL*, *GLIS1*, *API5*, and *HILPDA*, which play significant roles in meat quality, adipogenesis and metabolic diseases (Arce‐Recinos et al., 2021; Bernal Rubio et al., 2015; DiStefano et al., 2016; Niu et al., 2016; Peng et al., 2015). The *Ferritin Light Chain* (*FTL*) gene has been associated with pork redness, a crucial aspect of eating quality (Bernal Rubio et al., 2015). The *GLIS Family Zinc Finger 1* (*GLIS1*) gene was identified as a pro‐adipogenic factor, which may be fundamentally involved in differentiating mesodermal cells during fetal development. In sheep, this gene may influence fat deposition in their tails, while its effect in pigs remains unknown (Arce‐Recinos et al., 2021). The *Apoptosis Inhibitor 5* (*API5*) gene has implications for various human diseases, including diabetes (Peng et al., 2015). However, in pigs, *API5* may serve a significant function in developing skeletal muscle, functioning as a housekeeping gene (Niu et al., 2016). Additionally, *HILPDA* induces triglyceride accumulation in hepatocytes (de la Rosa Rodriguez et al., 2021; DiStefano et al., 2015). *HILPDA* levels rise in response to multiple factors, such as hypoxia, *β*‐adrenergic activation and FAs (Deng et al., 2023). The SNP significantly associated with linolenic acid variation was SSC8:38763511. This SNP is associated with the gene *SLAIN2*, which is located in a *cis‐*eQTL region. *SLAIN Motif Family Member 2* (*SLAIN2*) is a growth factor with high expression level in muscle tissue. *SLAIN2* has also been associated with weight in broilers (Tarsani et al., 2019).

All of the methodological precautions, such as the use of a stringent FDR correction, were taken to ensure the reliability and biological consistency of our findings. The results revealed novel and biologically meaningful insights into gene expression regulation that had not been previously described in this context. The integration of motif analysis and TFs enabled a deeper exploration of regulatory mechanisms. These preliminary findings, although constrained by limited sample size, present biologically coherent patterns and provide a starting point to guide future research, helping to refine target selection and experimental design in larger populations. Therefore, this study not only contributes new biological knowledge but also offers a valuable foundation for further studies.

## CONCLUSIONS

In this study, we identified TFs related to lipid metabolism and stress regulation, which are also involved in metabolic disease pathways. In addition to the TFs, we identified important candidate genes associated with FA composition variation that affect pork quality and potentially human health. While these genes and genomic factors may serve as key regulators in metabolic disease response and lipid metabolism, further studies using larger datasets are needed to validate these results. Our findings provide preliminary insights into the gene expression mechanisms associated with lipid metabolism, pork quality and FA composition variation in pigs.

## AUTHOR CONTRIBUTIONS

Conceptualization, M.C.D., F.N.C., L.F.B. (Benfica), S.L.F., L.E.N., B.P.M.S., B.S.V., A.S.M.C., and L.F.B. (Brito); methodology, M.C.D., C.S.O., I.S.G., A.O.R., F.A.O.F., B.S.V., L.F.B. (Benfica), and A.S.M.C.; software, M.C.D., A.O.R., F.A.O.F.; writing – original draft preparation, M.C.D., L.F.B. (Benfica), S.L.F., A.S.M.C., and L.F.B. (Brito); writing – review and editing, M.C.D., B.P.M.S., A.S.M.C., and L.F.B. (Brito); supervision, A.S.M.C., and L.F.B. (Brito); funding acquisition, A.S.M.C. All authors have read and agreed to the published version of the manuscript.

## FUNDING INFORMATION

This research was funded by the São Paulo Research Foundation (FAPESP, grant numbers 2017/25180‐2, 2023/07931‐1, 2023/06442‐7 and 2022/08142‐8) and the Brazilian National Council for Scientific and Technological Development that provided a researcher fellowship to A. S. M. Cesar (303165/2022‐7). This study was financed in part by the Coordenação de Aperfeiçoamento de Pessoal de Nível Superior – Brazil – Finance Code 001.

## CONFLICT OF INTEREST STATEMENT

The authors declare no conflicts of interest.

## ETHICS STATEMENT

The animal study protocol was approved by the Animal Care and Use Committee from the Luiz de Queiroz College of Agriculture (University of São Paulo, Piracicaba, SP, Brazil; protocol number 2018.5.1787.11.6) and the Ethics Committee on Animal Use (number 2018‐28).

## Supporting information


Figure S1.

Table S1.

Table S2.

Table S3.

Table S4.

Table S5.

Table S6.

## Data Availability

The dataset used is available in the European Nucleotide Archive repository (EMBL‐EBI), under the accession PRJEB52629 (www.ebi.ac.uk/ena/data/view/PRJEB52629). Further inquiries can be directed to the corresponding author.
