# Supplementary material for: Transcription factors and candidate functional SNPs associated with variation in fatty acid composition from skeletal muscle of pigs
Source: Anim Genet. 2025 Oct 11;56(5):e70051. doi: 10.1111/age.70051 (PMC12514662; doi:10.1111/age.70051)

Figure S1. The most significantly enriched Motif Logos from the STREME Suite for the 23 Hotspots

HOTSPOTS 1 (3_102983783)

E-value: 1.1e-008


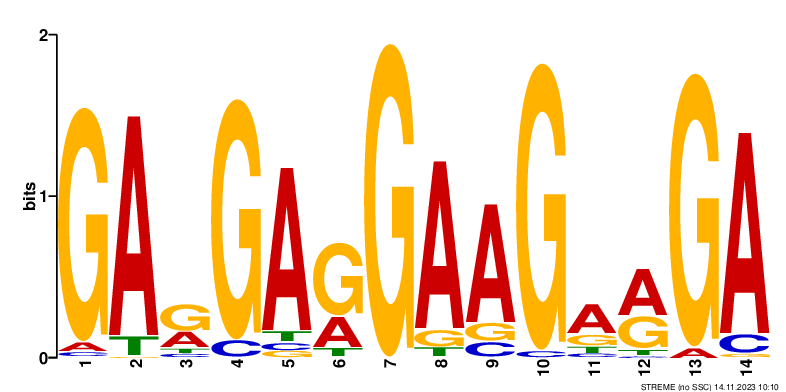


HOTSPOTS 2 (14_29337834)

E-value: 1.9e-009


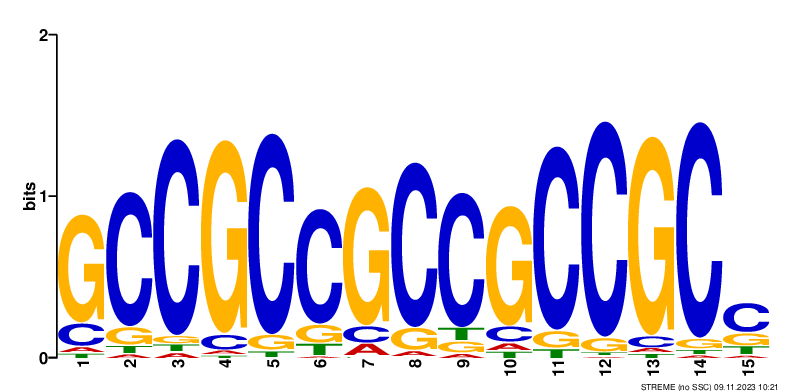


HOTSPOTS 3 (8_87522621)

E-value: 6.3e-006


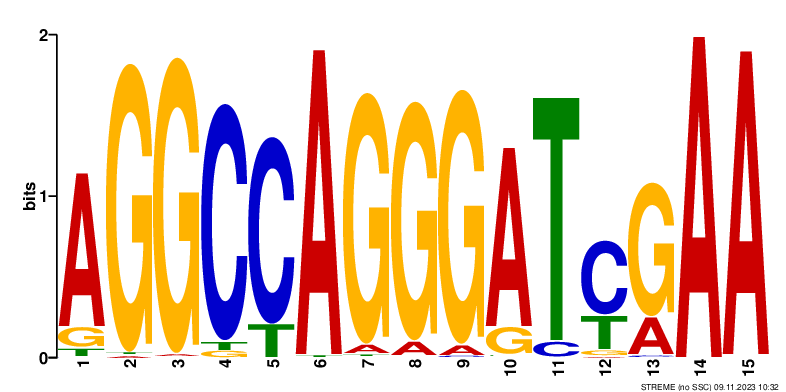


HOTSPOTS 4 (11_22233646)

E-value: 7.9e-010


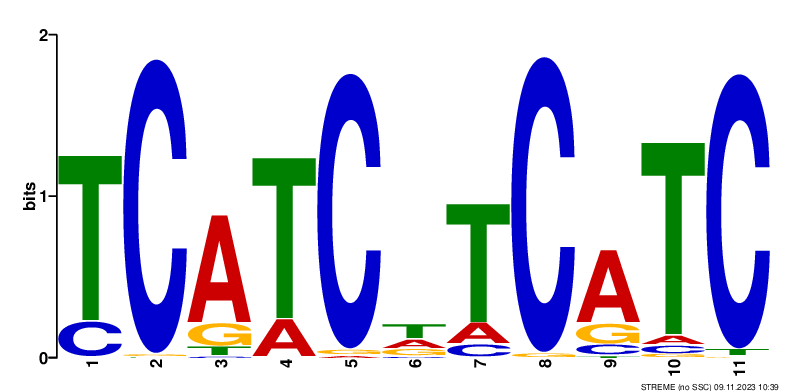


HOTSPOTS 5 (6_49404697)

E-value: 1.6e-008


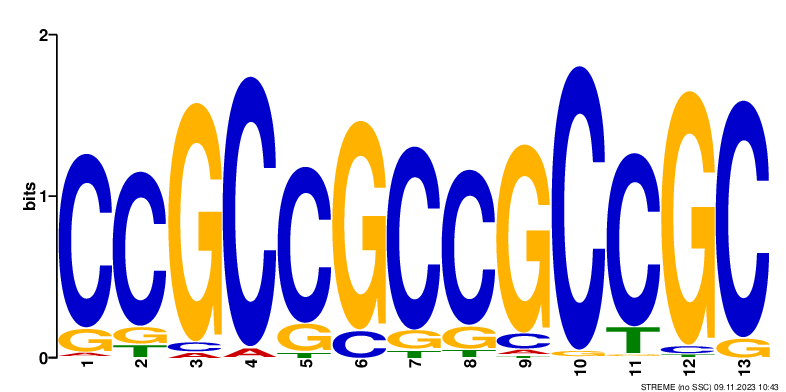


HOTSPOTS 6 (1_66388768)

E-value: 1.3e-010


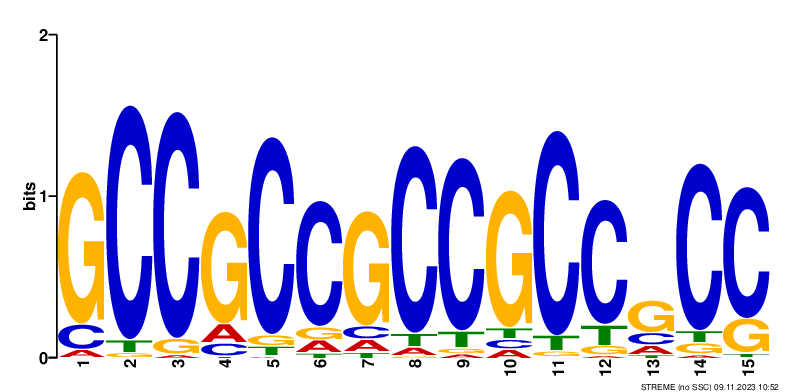


HOTSPOTS 7 (2_67926641)

E-value: 4.3e-006


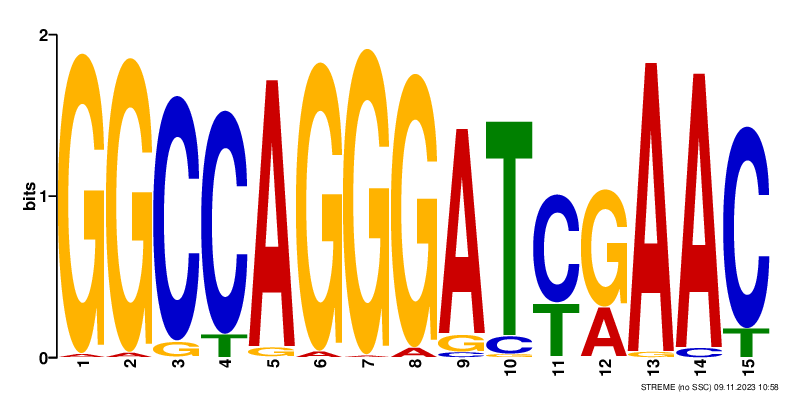


HOTSPOTS 8 (2_67926616)

E-value: 3.6e-007


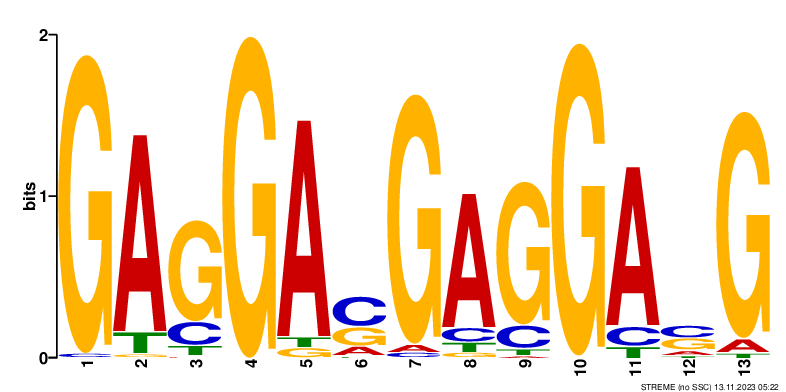


HOTSPOTS 9 (6_93742864)

E-value: 4.1e-010


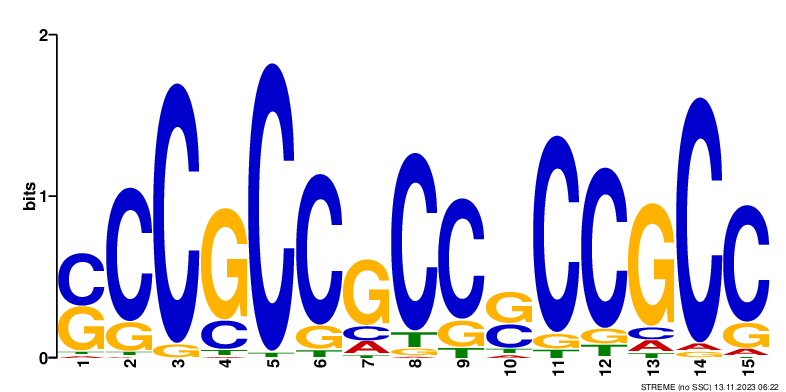


HOTSPOTS 10 (9_72368773)

E-value: 9.0e-008


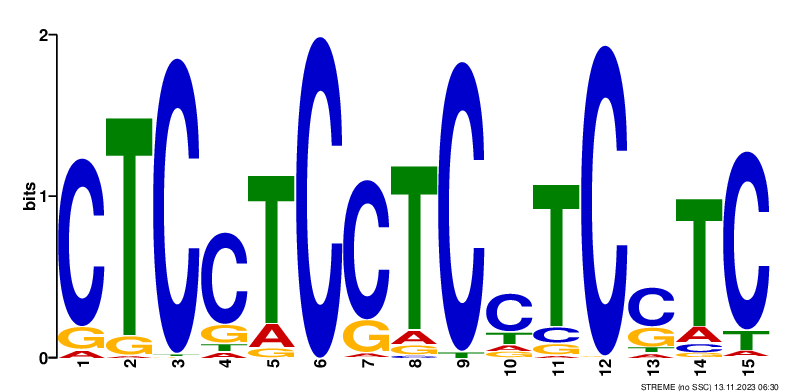


HOTSPOTS 11 (11_22233645)

E-value: 1.2e-006


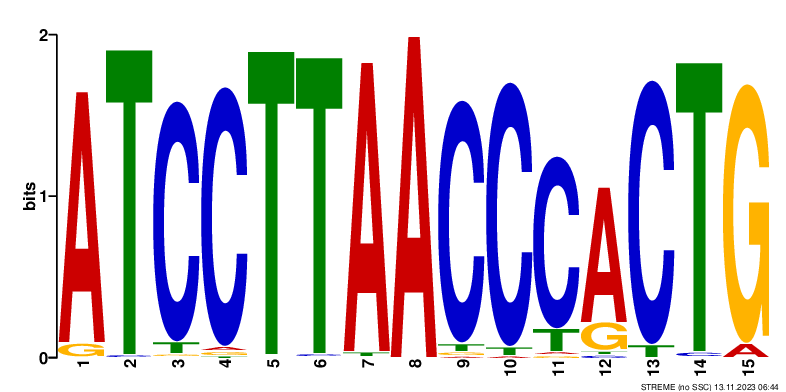


HOTSPOTS 12 (12_55140463)

E-value: 6.7e-008


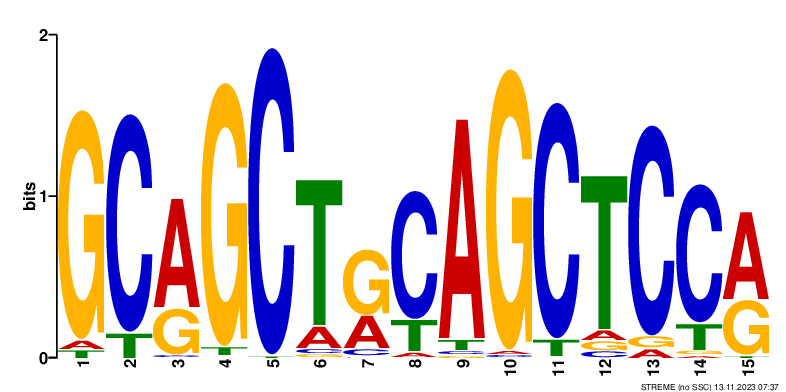


HOTSPOTS 13 (12_55141850)

E-value: 6.7e-008


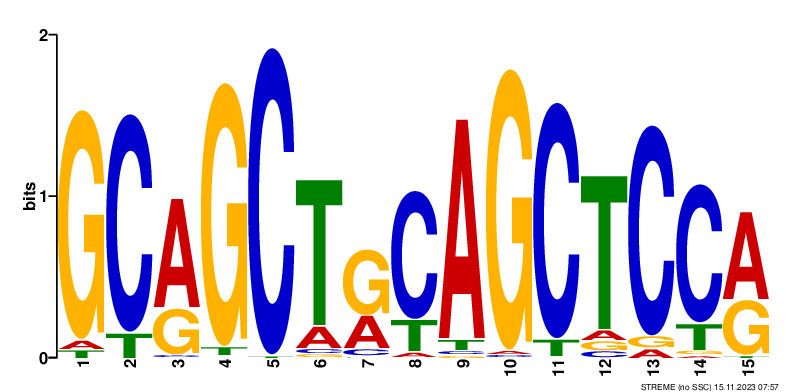


HOTSPOTS 14 (12_55199008)

E-value: 6.7e-008


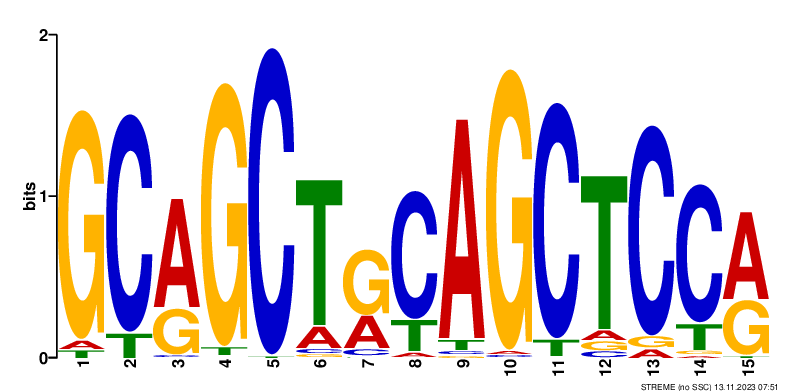


HOTSPOTS 15 (1_189152166)

E-value: 8.0e-008


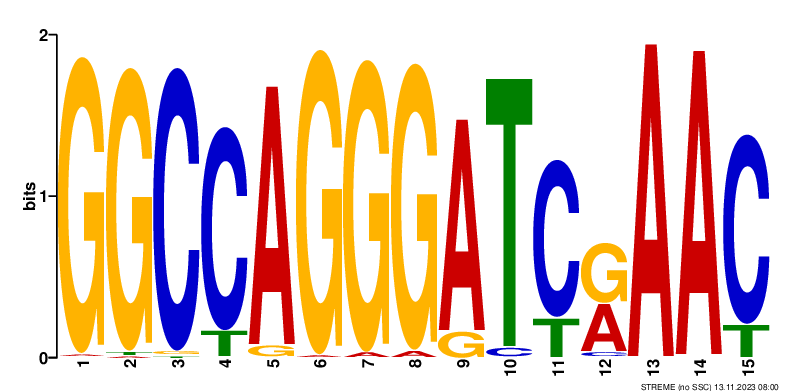


HOTSPOTS 16 (9_11163944)

E-value: 5.2e-009


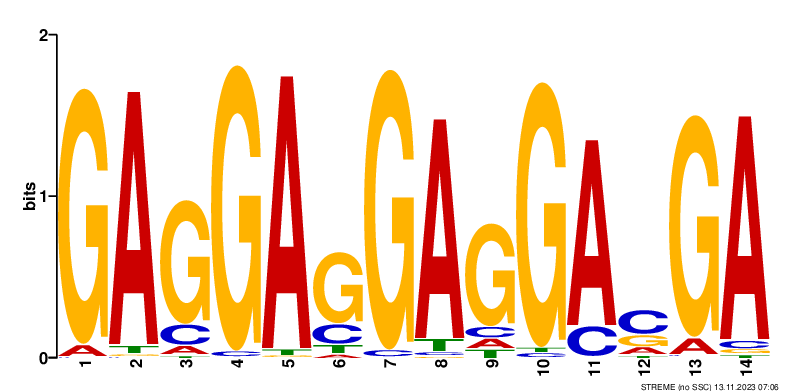


HOTSPOTS 17 (9_64780547)

E-value: 6.5e-013


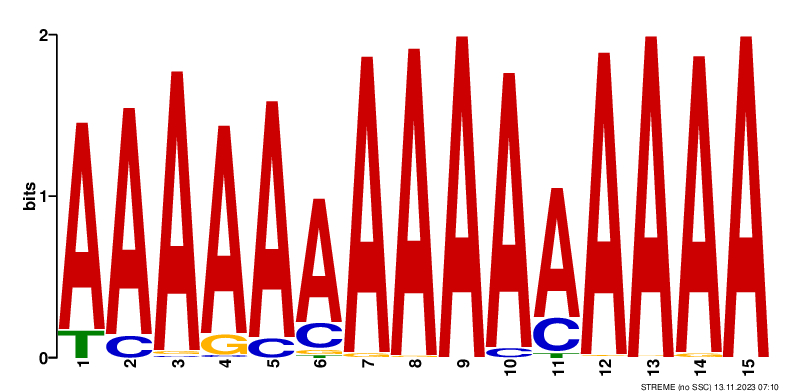


HOTSPOTS 18 (12_55138211)

E-value: 2.5e-007


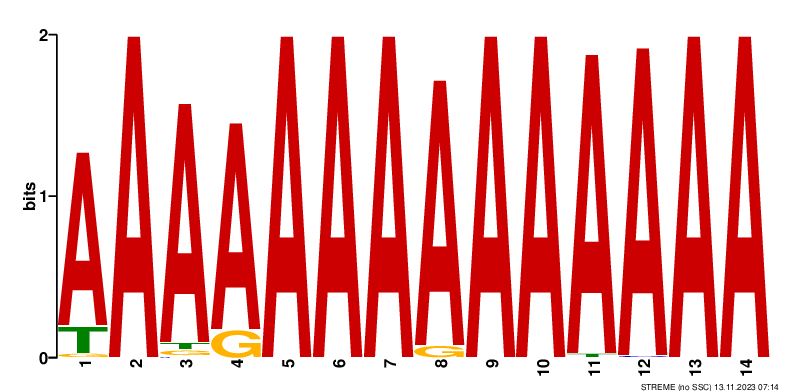


HOTSPOTS 19 (17_41468872)

E-value: 5.2e-005


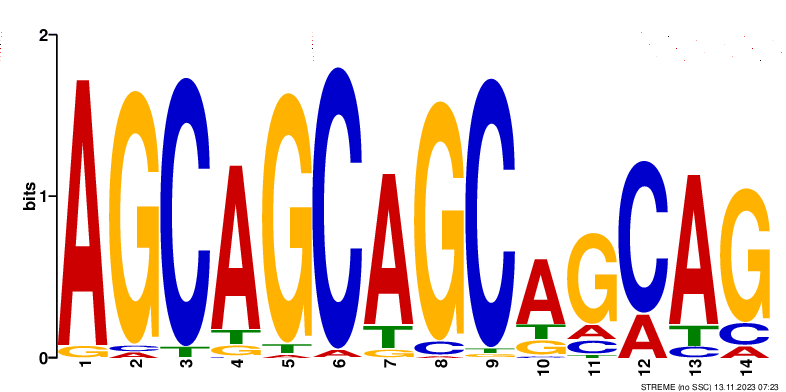


HOTSPOTS 20 (8_96887837)

E-value: 2.8e-007


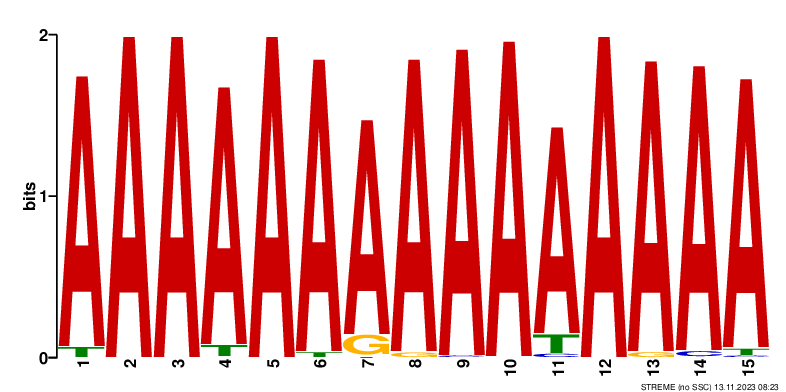


HOTSPOTS 21 (12_54832473)

E-value: 1.6e-005


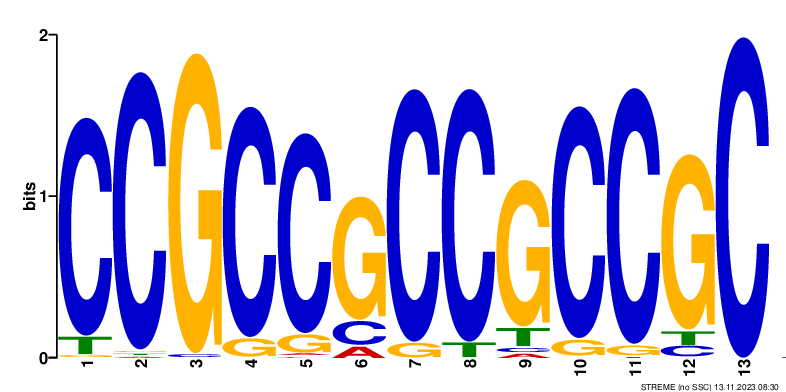


HOTSPOTS 22 (12_54936914)

E-value: 1.8e-006


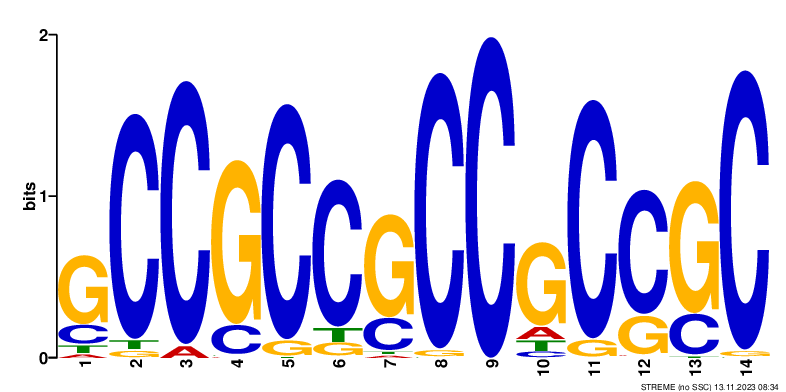


HOTSPOTS 23 (2_8444101)

E-value: 2.5e-005


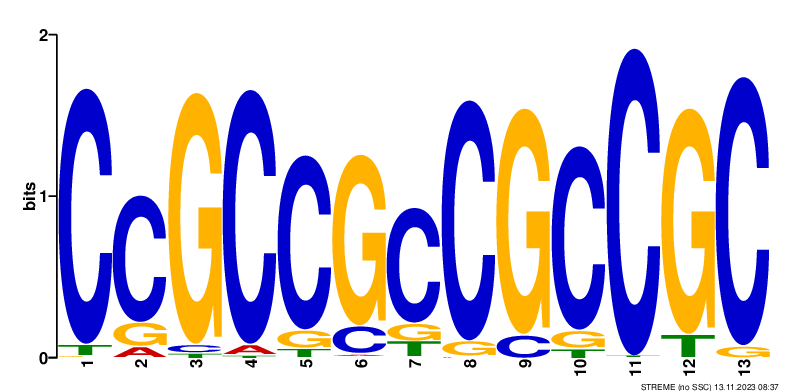

Supplement: Supplementary file 1 — Figure S1. [file AGE-56-0-s001.docx]
