# Supplementary material for: Transcription factors and candidate functional SNPs associated with variation in fatty acid composition from skeletal muscle of pigs
Source: Anim Genet. 2025 Oct 11;56(5):e70051. doi: 10.1111/age.70051 (PMC12514662; doi:10.1111/age.70051)
Supplement: Supplementary file 5 — Table S4. [file AGE-56-0-s007.docx]

**Supplementary Table S4**. Kyoto encyclopedia of genes and genomes (KEGG) pathway enrichment analysis for cis-eQTL regulated genes by using DAVID software

| Term | Count | P-value |
| --- | --- | --- |
| ssc05168:Herpes simplex virus 1 infection | 61 | <0.001 |
| ssc01100:Metabolic pathways | 162 | <0.001 |
| ssc00562:Inositol phosphate metabolism | 15 | <0.001 |
| ssc04070:Phosphatidylinositol signaling system | 16 | 0.002 |
| ssc00982:Drug metabolism - cytochrome P450 | 11 | 0.003 |
| ssc03082:ATP-dependent chromatin remodeling | 18 | 0.003 |
| ssc00750:Vitamin B6 metabolism | 4 | 0.015 |
| ssc00980:Metabolism of xenobiotics by cytochrome P450 | 10 | 0.021 |
| ssc05164:Influenza A | 20 | 0.023 |
| ssc03022:Basal transcription factors | 8 | 0.031 |
| ssc00511:Other glycan degradation | 5 | 0.035 |
| ssc04928:Parathyroid hormone synthesis, secretion and action | 14 | 0.036 |
| ssc04814:Motor proteins | 21 | 0.043 |
| ssc00350:Tyrosine metabolism | 7 | 0.045 |
| ssc04140:Autophagy - animal | 19 | 0.047 |

The label "Term" lists the names of the KEGG pathways related to fatty acid and lipid metabolism; The label "Count" indicates the number of genes involved in each KEGG pathway; The label "P-value" represents the statistical significance for the enrichment of each KEGG pathway.
