# Supplementary material for: Transcription factors and candidate functional SNPs associated with variation in fatty acid composition from skeletal muscle of pigs
Source: Anim Genet. 2025 Oct 11;56(5):e70051. doi: 10.1111/age.70051 (PMC12514662; doi:10.1111/age.70051)
Supplement: Supplementary file 6 — Table S5. [file AGE-56-0-s005.docx]

**Supplementary Table S5**. Kyoto encyclopedia of genes and genomes (KEGG) pathway enrichment analysis for trans-eQTL regulated genes by using DAVID software.

| Term | Count | PValue |
| --- | --- | --- |
| ssc01100:Metabolic pathways | 958 | <0.001 |
| ssc04144:Endocytosis | 170 | <0.001 |
| ssc04360:Axon guidance | 135 | <0.001 |
| ssc05200:Pathways in cancer | 329 | <0.001 |
| ssc04015:Rap1 signaling pathway | 151 | <0.001 |
| ssc04520:Adherens junction | 77 | <0.001 |
| ssc04510:Focal adhesion | 140 | <0.001 |
| ssc01200:Carbon metabolism | 87 | <0.001 |
| ssc04071:Sphingolipid signaling pathway | 90 | <0.001 |
| ssc05205:Proteoglycans in cancer | 140 | <0.001 |
| ssc04151:PI3K-Akt signaling pathway | 220 | <0.001 |
| ssc05166:Human T-cell leukemia virus 1 infection | 150 | <0.001 |
| ssc04611:Platelet activation | 90 | <0.001 |
| ssc04922:Glucagon signaling pathway | 77 | <0.001 |
| ssc05415:Diabetic cardiomyopathy | 138 | <0.001 |
| ssc04070:Phosphatidylinositol signaling system | 73 | <0.001 |
| ssc04933:AGE-RAGE signaling pathway in diabetic complications | 76 | <0.001 |
| ssc04072:Phospholipase D signaling pathway | 105 | <0.001 |
| ssc05165:Human papillomavirus infection | 205 | <0.001 |
| ssc04350:TGF-beta signaling pathway | 81 | <0.001 |
| ssc04810:Regulation of actin cytoskeleton | 149 | <0.001 |
| ssc05163:Human cytomegalovirus infection | 149 | <0.001 |
| ssc04714:Thermogenesis | 151 | <0.001 |
| ssc04152:AMPK signaling pathway | 88 | <0.001 |
| ssc05132:Salmonella infection | 162 | <0.001 |
| ssc04371:Apelin signaling pathway | 97 | <0.001 |
| ssc04261:Adrenergic signaling in cardiomyocytes | 103 | <0.001 |
| ssc05100:Bacterial invasion of epithelial cells | 60 | <0.001 |
| ssc04066:HIF-1 signaling pathway | 79 | <0.001 |
| ssc04062:Chemokine signaling pathway | 121 | <0.001 |
| ssc04140:Autophagy - animal | 111 | <0.001 |
| ssc05418:Fluid shear stress and atherosclerosis | 96 | <0.001 |
| ssc04931:Insulin resistance | 79 | <0.001 |
| ssc04666:Fc gamma R-mediated phagocytosis | 69 | <0.001 |
| ssc00562:Inositol phosphate metabolism | 55 | <0.001 |
| ssc04142:Lysosome | 90 | <0.001 |
| ssc05145:Toxoplasmosis | 76 | 6.23E-07 |
| ssc04022:cGMP-PKG signaling pathway | 111 | 6.52E-07 |
| ssc05142:Chagas disease | 77 | 7.44E-07 |
| ssc04014:Ras signaling pathway | 149 | 7.81E-07 |
| ssc01521:EGFR tyrosine kinase inhibitor resistance | 59 | 8.29E-07 |
| ssc04910:Insulin signaling pathway | 93 | 9.15E-07 |
| ssc04218:Cellular senescence | 103 | 1.12E-06 |
| ssc04921:Oxytocin signaling pathway | 100 | 1.29E-06 |
| ssc04010:MAPK signaling pathway | 180 | 1.40E-06 |
| ssc05146:Amoebiasis | 71 | 1.53E-06 |
| ssc00510:N-Glycan biosynthesis | 43 | 1.57E-06 |
| ssc04110:Cell cycle | 102 | 2.50E-06 |
| ssc04020:Calcium signaling pathway | 158 | 2.57E-06 |
| ssc04068:FoxO signaling pathway | 88 | 3.58E-06 |
| ssc00600:Sphingolipid metabolism | 43 | 3.84E-06 |
| ssc04390:Hippo signaling pathway | 102 | 3.88E-06 |
| ssc04211:Longevity regulating pathway | 64 | 4.79E-06 |
| ssc05133:Pertussis | 55 | 5.25E-06 |
| ssc03040:Spliceosome | 84 | 5.91E-06 |
| ssc05168:Herpes simplex virus 1 infection | 208 | 6.33E-06 |
| ssc04925:Aldosterone synthesis and secretion | 66 | 6.78E-06 |
| ssc05417:Lipid and atherosclerosis | 135 | 8.34E-06 |
| ssc03250:Viral life cycle - HIV-1 | 43 | 8.86E-06 |
| ssc04213:Longevity regulating pathway - multiple species | 46 | 9.72E-06 |
| ssc04934:Cushing syndrome | 98 | 9.74E-06 |
| ssc04919:Thyroid hormone signaling pathway | 81 | 1.09E-05 |
| ssc04926:Relaxin signaling pathway | 86 | 1.17E-05 |
| ssc04670:Leukocyte transendothelial migration | 78 | 1.25E-05 |
| ssc04927:Cortisol synthesis and secretion | 47 | 1.27E-05 |
| ssc00020:Citrate cycle (TCA cycle) | 26 | 1.41E-05 |
| ssc04141:Protein processing in endoplasmic reticulum | 106 | 1.80E-05 |
| ssc05208:Chemical carcinogenesis - reactive oxygen species | 139 | 1.95E-05 |
| ssc04114:Oocyte meiosis | 80 | 2.49E-05 |
| ssc05231:Choline metabolism in cancer | 66 | 3.43E-05 |
| ssc05230:Central carbon metabolism in cancer | 49 | 3.90E-05 |
| ssc01212:Fatty acid metabolism | 43 | 4.00E-05 |
| ssc00010:Glycolysis / Gluconeogenesis | 46 | 4.01E-05 |
| ssc05167:Kaposi sarcoma-associated herpesvirus infection | 121 | 4.26E-05 |
| ssc05225:Hepatocellular carcinoma | 103 | 4.51E-05 |
| ssc04145:Phagosome | 93 | 4.93E-05 |
| ssc04932:Non-alcoholic fatty liver disease | 100 | 5.39E-05 |
| ssc04330:Notch signaling pathway | 45 | 6.26E-05 |
| ssc05203:Viral carcinogenesis | 118 | 7.08E-05 |
| ssc00230:Purine metabolism | 83 | 1.13E-04 |
| ssc04530:Tight junction | 103 | 1.28E-04 |
| ssc05135:Yersinia infection | 91 | 1.32E-04 |
| ssc04916:Melanogenesis | 66 | 1.44E-04 |
| ssc04730:Long-term depression | 43 | 1.50E-04 |
| ssc05170:Human immunodeficiency virus 1 infection | 131 | 1.57E-04 |
| ssc04722:Neurotrophin signaling pathway | 77 | 1.60E-04 |
| ssc05221:Acute myeloid leukemia | 47 | 1.66E-04 |
| ssc03013:Nucleocytoplasmic transport | 70 | 2.07E-04 |
| ssc04750:Inflammatory mediator regulation of TRP channels | 71 | 2.24E-04 |
| ssc01250:Biosynthesis of nucleotide sugars | 30 | 2.30E-04 |
| ssc04659:Th17 cell differentiation | 72 | 2.43E-04 |
| ssc05212:Pancreatic cancer | 51 | 2.93E-04 |
| ssc00520:Amino sugar and nucleotide sugar metabolism | 37 | 3.09E-04 |
| ssc04924:Renin secretion | 48 | 3.32E-04 |
| ssc04540:Gap junction | 57 | 3.57E-04 |
| ssc05171:Coronavirus disease - COVID-19 | 146 | 3.89E-04 |
| ssc04625:C-type lectin receptor signaling pathway | 68 | 3.98E-04 |
| ssc05215:Prostate cancer | 63 | 4.02E-04 |
| ssc04725:Cholinergic synapse | 72 | 5.25E-04 |
| ssc04923:Regulation of lipolysis in adipocytes | 40 | 5.29E-04 |
| ssc00190:Oxidative phosphorylation | 85 | 5.39E-04 |
| ssc04713:Circadian entrainment | 64 | 6.52E-04 |
| ssc05410:Hypertrophic cardiomyopathy | 59 | 6.63E-04 |
| ssc05152:Tuberculosis | 110 | 7.56E-04 |
| ssc00513:Various types of N-glycan biosynthesis | 32 | 7.78E-04 |
| ssc04928:Parathyroid hormone synthesis, secretion and action | 67 | 8.01E-04 |
| ssc04370:VEGF signaling pathway | 40 | 9.18E-04 |
| ssc05140:Leishmaniasis | 49 | 9.85E-04 |
| ssc04658:Th1 and Th2 cell differentiation | 60 | 0.00107 |
| ssc04024:cAMP signaling pathway | 130 | 0.001194 |
| ssc04136:Autophagy - other | 25 | 0.001245 |
| ssc00640:Propanoate metabolism | 25 | 0.001245 |
| ssc00310:Lysine degradation | 43 | 0.001333 |
| ssc04912:GnRH signaling pathway | 58 | 0.00137 |
| ssc04914:Progesterone-mediated oocyte maturation | 59 | 0.001467 |
| ssc04120:Ubiquitin mediated proteolysis | 87 | 0.001493 |
| ssc00030:Pentose phosphate pathway | 23 | 0.001547 |
| ssc04380:Osteoclast differentiation | 80 | 0.001552 |
| ssc05222:Small cell lung cancer | 60 | 0.001565 |
| ssc04935:Growth hormone synthesis, secretion and action | 73 | 0.001577 |
| ssc00532:Glycosaminoglycan biosynthesis - chondroitin sulfate / dermatan sulfate | 18 | 0.001585 |
| ssc04310:Wnt signaling pathway | 101 | 0.001722 |
| ssc04270:Vascular smooth muscle contraction | 83 | 0.001739 |
| ssc03320:PPAR signaling pathway | 51 | 0.001809 |
| ssc04668:TNF signaling pathway | 69 | 0.001814 |
| ssc00604:Glycosphingolipid biosynthesis - ganglio series | 14 | 0.00187 |
| ssc04640:Hematopoietic cell lineage | 57 | 0.001876 |
| ssc05214:Glioma | 47 | 0.001976 |
| ssc03015:mRNA surveillance pathway | 58 | 0.001999 |
| ssc01232:Nucleotide metabolism | 53 | 0.002099 |
| ssc00620:Pyruvate metabolism | 31 | 0.002207 |
| ssc05235:PD-L1 expression and PD-1 checkpoint pathway in cancer | 60 | 0.002251 |
| ssc04961:Endocrine and other factor-regulated calcium reabsorption | 35 | 0.002274 |
| ssc00564:Glycerophospholipid metabolism | 61 | 0.002379 |
| ssc04936:Alcoholic liver disease | 84 | 0.002402 |
| ssc04392:Hippo signaling pathway - multiple species | 22 | 0.002532 |
| ssc05161:Hepatitis B | 98 | 0.002681 |
| ssc03018:RNA degradation | 51 | 0.002693 |
| ssc05223:Non-small cell lung cancer | 46 | 0.00277 |
| ssc04920:Adipocytokine signaling pathway | 47 | 0.002995 |
| ssc01230:Biosynthesis of amino acids | 47 | 0.002995 |
| ssc05412:Arrhythmogenic right ventricular cardiomyopathy | 49 | 0.003456 |
| ssc03010:Ribosome | 88 | 0.00353 |
| ssc03082:ATP-dependent chromatin remodeling | 71 | 0.003701 |
| ssc04210:Apoptosis | 81 | 0.003827 |
| ssc00051:Fructose and mannose metabolism | 24 | 0.003979 |
| ssc01522:Endocrine resistance | 58 | 0.004043 |
| ssc05414:Dilated cardiomyopathy | 59 | 0.00424 |
| ssc04918:Thyroid hormone synthesis | 47 | 0.004435 |
| ssc04724:Glutamatergic synapse | 69 | 0.004636 |
| ssc05220:Chronic myeloid leukemia | 48 | 0.004728 |
| ssc00500:Starch and sucrose metabolism | 25 | 0.004779 |
| ssc01040:Biosynthesis of unsaturated fatty acids | 22 | 0.005116 |
| ssc04512:ECM-receptor interaction | 56 | 0.005129 |
| ssc04662:B cell receptor signaling pathway | 50 | 0.005319 |
| ssc00630:Glyoxylate and dicarboxylate metabolism | 23 | 0.006191 |
| ssc04911:Insulin secretion | 53 | 0.006208 |
| ssc04971:Gastric acid secretion | 47 | 0.006425 |
| ssc04146:Peroxisome | 54 | 0.006503 |
| ssc05164:Influenza A | 94 | 0.006526 |
| ssc00062:Fatty acid elongation | 20 | 0.006572 |
| ssc04720:Long-term potentiation | 42 | 0.006854 |
| ssc04130:SNARE interactions in vesicular transport | 24 | 0.007309 |
| ssc05202:Transcriptional misregulation in cancer | 105 | 0.00762 |
| ssc05213:Endometrial cancer | 38 | 0.00768 |
| ssc04012:ErbB signaling pathway | 52 | 0.00824 |
| ssc04550:Signaling pathways regulating pluripotency of stem cells | 83 | 0.008618 |
| ssc05022:Pathways of neurodegeneration - multiple diseases | 250 | 0.008836 |
| ssc04340:Hedgehog signaling pathway | 35 | 0.009207 |
| ssc04970:Salivary secretion | 55 | 0.009297 |
| ssc04064:NF-kappa B signaling pathway | 64 | 0.009326 |
| ssc04929:GnRH secretion | 41 | 0.009386 |
| ssc04728:Dopaminergic synapse | 76 | 0.009598 |
| ssc05211:Renal cell carcinoma | 43 | 0.010509 |
| ssc04660:T cell receptor signaling pathway | 73 | 0.011565 |
| ssc05210:Colorectal cancer | 54 | 0.012117 |
| ssc04115:p53 signaling pathway | 46 | 0.01215 |
| ssc00280:Valine, leucine and isoleucine degradation | 34 | 0.012841 |
| ssc00071:Fatty acid degradation | 29 | 0.013167 |
| ssc04962:Vasopressin-regulated water reabsorption | 29 | 0.013167 |
| ssc01240:Biosynthesis of cofactors | 82 | 0.013463 |
| ssc03083:Polycomb repressive complex | 50 | 0.014228 |
| ssc04917:Prolactin signaling pathway | 44 | 0.015432 |
| ssc05010:Alzheimer disease | 203 | 0.016034 |
| ssc00052:Galactose metabolism | 22 | 0.016532 |
| ssc04664:Fc epsilon RI signaling pathway | 41 | 0.018806 |
| ssc04723:Retrograde endocannabinoid signaling | 84 | 0.021116 |
| ssc00561:Glycerolipid metabolism | 38 | 0.022955 |
| ssc04621:NOD-like receptor signaling pathway | 97 | 0.023237 |
| ssc04915:Estrogen signaling pathway | 75 | 0.023707 |
| ssc05160:Hepatitis C | 88 | 0.026515 |
| ssc00511:Other glycan degradation | 14 | 0.028064 |
| ssc04814:Motor proteins | 102 | 0.028377 |
| ssc05016:Huntington disease | 163 | 0.028869 |
| ssc05226:Gastric cancer | 83 | 0.031033 |
| ssc04930:Type II diabetes mellitus | 30 | 0.031311 |
| ssc04217:Necroptosis | 87 | 0.031762 |
| ssc05323:Rheumatoid arthritis | 54 | 0.035133 |
| ssc04972:Pancreatic secretion | 57 | 0.036737 |
| ssc05224:Breast cancer | 80 | 0.044178 |
| ssc04710:Circadian rhythm | 23 | 0.044577 |
| ssc05218:Melanoma | 41 | 0.045502 |
| ssc04979:Cholesterol metabolism | 31 | 0.045812 |
| ssc04137:Mitophagy - animal | 42 | 0.046479 |
| ssc00270:Cysteine and methionine metabolism | 33 | 0.048988 |
| ssc05219:Bladder cancer | 25 | 0.049778 |
